# Pro-Inflammatory Flagellin Proteins of Prevalent Motile Commensal Bacteria Are Variably Abundant in the Intestinal Microbiome of Elderly Humans

**DOI:** 10.1371/journal.pone.0068919

**Published:** 2013-07-23

**Authors:** B. Anne Neville, Paul O. Sheridan, Hugh M. B. Harris, Simone Coughlan, Harry J. Flint, Sylvia H. Duncan, Ian B. Jeffery, Marcus J. Claesson, R. Paul Ross, Karen P. Scott, Paul W. O'Toole

**Affiliations:** 1 Department of Microbiology, University College Cork, Cork, Ireland; 2 Rowett Institute of Nutrition and Health, University of Aberdeen, Bucksburn, Aberdeen, United Kingdom; 3 Teagasc Moorepark Food Research Centre, Fermoy, County Cork, Ireland; University of Hyderabad, India

## Abstract

Some *Eubacterium* and *Roseburia* species are among the most prevalent motile bacteria present in the intestinal microbiota of healthy adults. These flagellate species contribute “cell motility” category genes to the intestinal microbiome and flagellin proteins to the intestinal proteome. We reviewed and revised the annotation of motility genes in the genomes of six *Eubacterium* and *Roseburia* species that occur in the human intestinal microbiota and examined their respective locus organization by comparative genomics. Motility gene order was generally conserved across these loci. Five of these species harbored multiple genes for predicted flagellins. Flagellin proteins were isolated from *R. inulinivorans* strain A2-194 and from *E. rectale* strains A1-86 and M104/1. The amino-termini sequences of the *R. inulinivorans* and *E. rectale* A1-86 proteins were almost identical. These protein preparations stimulated secretion of interleukin-8 (IL-8) from human intestinal epithelial cell lines, suggesting that these flagellins were pro-inflammatory. Flagellins from the other four species were predicted to be pro-inflammatory on the basis of alignment to the consensus sequence of pro-inflammatory flagellins from the β- and γ- proteobacteria. Many *fliC* genes were deduced to be under the control of σ^28^. The relative abundance of the target *Eubacterium* and *Roseburia* species varied across shotgun metagenomes from 27 elderly individuals. Genes involved in the flagellum biogenesis pathways of these species were variably abundant in these metagenomes, suggesting that the current depth of coverage used for metagenomic sequencing (3.13–4.79 Gb total sequence in our study) insufficiently captures the functional diversity of genomes present at low (≤1%) relative abundance. *E. rectale* and *R. inulinivorans* thus appear to synthesize complex flagella composed of flagellin proteins that stimulate IL-8 production. A greater depth of sequencing, improved evenness of sequencing and improved metagenome assembly from short reads will be required to facilitate *in silico* analyses of complete complex biochemical pathways for low-abundance target species from shotgun metagenomes.

## Introduction

The mammalian colon is one of the most densely populated microbial ecosystems known [1]. The microorganisms that occupy this niche, which are collectively known as the colonic microbiota, can influence the health and well-being of the host by affecting physiological and immune functions [2]–[7]. In particular, microbial metabolites, structural molecules and released cellular components are potential antigens and microbe-associated molecular patterns (MAMPs) that may stimulate the immune system [8]. The collection of genomes from the members of a microbial community is known as a microbiome. The genes and functions encoded by the intestinal microbiome therefore govern which bacterial and food-derived immunomodulatory molecules are likely to be present in the intestine.

The genomes of bacteria from many different lineages encode genes for flagellum assembly, and the distribution of these genes among bacteria has been considered previously [9], [10]. Many genes are required for the synthesis of a functional flagellum [10], [11]. Flagellin is the major structural protein in the flagellar filaments of motile bacteria [12]. Flagellins and the genes encoding them are variably abundant in the intestines [13]–[15] and the “cell motility” category has been reported as a low-abundance microbial function in this niche [16], [17]. Motile bacteria bear significant immunostimulatory potential because humans and other animals harbor cell-surface and cytoplasmic pattern recognition receptors which respond to extra- and intra- cellular flagellin molecules respectively [18]–[20].

Particular motile *Eubacterium* and *Roseburia* species are among the most prevalent bacterial species in the human intestinal microbiota [16], [21]–[24]. These commensals are also notable as producers of the short chain fatty acid, butyrate, in the gut [25], [26]. To date, the genetic basis for flagellum biogenesis among these *Eubacterium* and *Roseburia* species has not been formally characterized, nor has the potential immune response to their flagellin proteins been established. However, it is known that heat-killed *Eubacterium rectale* cells can induce nuclear factor-κB (NF-κB) by signalling through TLR2 and TLR5 [13]. Conditioned media from *Roseburia* cultures significantly stimulated and enhanced NF-κB activation in HT-29 and Caco-2 cells, while conditioned medium from *E. rectale* had an inhibitory effect on NF-κB activation [27]. The authors of this study attributed the immunomodulatory properties of these strains to flagellin and also to butyrate production, (which was shown to be positively correlated with NF-κB activity in TNF-α treated cell lines) [27]. Furthermore, flagellin proteins from members of *Clostridium* cluster XIV, which includes some of the species examined here, have been circumstantially implicated in the development of Crohn's disease and murine colitis [28], [29].

The genera *Roseburia* and *Eubacterium* are members of the phylum *Firmicutes* [30]. While the genus *Eubacterium* is large and heterogeneous, the genus *Roseburia* is small and homogeneous [31], [32]. The reclassification of *Eubacterium* species to other genera is quite common [33]. Indeed, *E. rectale* could be more appropriately classified as a *Roseburia* species on the basis of 16S rRNA gene analyses and phenotypic properties [31], but to date its classification and nomenclature have not been revised. Each of the *Roseburia* species isolated has been described as either flagellate or motile [31], [34]. Not all *Eubacterium* species are motile. Species for which motility has been reported include *E. acidaminophilum*, *E. cellulosolvens*, *E. combesii*, *E. desmolans*, *E. eligens*, *E. fissicatena*, *E. moniliforrme*, *E. multiforme*, *E. plautii*, *E. plexicaudatum*, *E. rectale*, *E. yurii* subsp. *yurii*, *E. yurii* subsp. *margaretiae* and *E. yurii* subsp. *schtitka* [35] and *E. siraeum* [35].

In this study, we describe the genetic basis for flagellum biogenesis in six of the motile *Eubacterium* and *Roseburia* species commonly isolated from the human gastrointestinal (GI) tract. We performed genome annotation and comparative genomics, focusing on the motility loci within the genomes of these species. The pro-inflammatory potential of their flagellin proteins was predicted *in silico*, and was also experimentally tested for flagellin proteins isolated from *E. rectale* and *R. inulinivorans* strains. We also aimed to determine if the present depth of sequencing used in the preparation of metagenome databases is sufficient to detect specific target genes from particular species. We focused on the detection of flagellum biogenesis genes from selected *Eubacterium* and *Roseburia* species in the datasets from an intestinal metagenomics project (ELDERMET) [34].

## Results

### Improvement of genome annotation and comparative genomics of *Eubacterium* and *Roseburia* motility loci

Initially the annotation of the genetic locus responsible for motility in each of these genomes was inspected, verified and improved as required (given that these annotations had previously been performed by automated means only). Open reading frames (ORFs) that had not been detected by the automated annotation system were included in our improved annotation, while genes with potential frame-shifts or contig breaks were identified. Frameshifts were corrected in the *fliJ* gene (ROSEINA2194_00946–00947) and the flagellar operon protein (FOP) (ROSEINA2194_00953–00954) genes in *R. inulinivorans*, *fliH* (ROSINTL182_07396–07395) in *R. intestinalis* and *fliF* (locus tag not assigned) in *R. hominis*. As these strains were shown to be motile, it is likely that these frameshifts are technical artefacts arising from sequencing or assembly errors. The primary motility locus was split over two contigs in the *R. intestinalis* genome assembly. The contig break occurred in the *flhA* gene.

**Figure 1:**
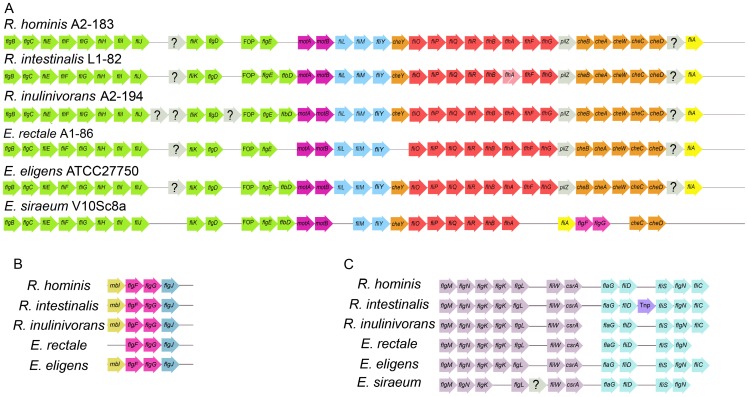
Gene order plot of major motility gene loci in *Eubacterium* and *Roseburia* genomes. Genes are represented by labelled arrows. Genes that are found consecutively at a single locus (A–C) are indicated by a horizontal line. The distances between the genes at these loci were modified in this schematic diagram so that homologous genes from different genomes could be aligned. Hypothetical genes are indicated by gray arrows with? symbols. A physical gap in the *R. intestinalis* genome assembly occurs in the *flhA* gene (Panel A, light red). A transposase gene (Tnp) is present between *fliD* and *fliS* in *R. intestinalis* (Panel C). The *flaG-flgN/fliC* gene cluster is not located immediately downstream of the *flgM-csrA* gene cluster in *R. inulinivorans* and *E. rectale* (Panel C). Colours were arbitrarily assigned to assist visual interpretation of gene rearrangements.

The gene content and genetic organization of the largest motility loci of six *Eubacterium* and *Roseburia* species were then compared (Figure 1, Table S1). Three motility loci, *flgB-fliA*, *flgM-flgN/fliC* and *mbl-flgJ* were identified in *E. rectale*, *E. eligens* and the three *Roseburia* genomes examined. The *flgB*-*fliA* locus of the *Lachnospiraceae* family contained at least 34 contiguous genes and spanned 30.5–31.5 kb (Figure 1, panel A, Table S1). The corresponding motility locus of *E. siraeum* V10Sc8a, a member species of the family *Ruminococcaceae* was smaller (∼26.3 kb) and included fewer genes (29) overall with a slightly different arrangement. Additionally, in the *E. siraeum* V10Sc8a genome, *flgF* and *flgG* were located within the *flgB-fliA* motility locus (Figure 1) and the genetic arrangement *mbl-flgF-flgG-flgJ* was not identified.

The arrangement of genes from *flgB* to *flgE* is generally well conserved in the *Eubacterium* and *Roseburia* genomes studied (Figure 1, panel A). Except for the *E. rectale* and *R. hominis* genomes, a *flbD* gene was present immediately downstream of *flgE* in each genome. The *motAB* gene pair was followed by *fliLMY* in each genome except the *E. siraeum* genome. The arrangement of genes between *fliO* and *pilZ* was conserved in *E. rectale*, *E. eligens* and all of the *Roseburia* genomes examined. This locus was interrupted by a *fliA-flgF-flgG* gene translocation in *E. siraeum*. A *cheY*-like chemotaxis gene immediately preceded the *fliO-pilZ* gene cluster in each genome except *E rectale* A1-86.

A set of five contiguous chemotaxis genes organized as *cheBAWCD* were located immediately downstream of *pilZ* in *E. rectale, E. eligens* and all of the *Roseburia* genomes studied. The equivalent *E. siraeum* V10Sc8a motility locus only contained the last two of these five chemotaxis genes. The *fliA* gene was the most distal gene at this locus for all species of the family *Lachnospiraceae* examined. In the *E. siraeum* genome, *cheD* is the most distal gene of this motility cluster and *fliA* is located between *flhA* and *flgF*.

A single *flgM-flgN/fliC* motility locus occurs in four of the six genomes studied (Figure 1, panel C; Table S1). In *R. inulinivoran*s A2-194 and *E. rectale* A1-86, this locus is divided into two separate gene clusters, the *flaG*-*flgN/fliC* gene cluster and the *flgM-csrA* gene cluster. Nevertheless, the genetic organization of each of these clusters is consistent with the organization of the single locus in the other genomes. Noteworthy features include the presence of two consecutive non-identical copies of *flgK* in five out of six genomes examined, the inclusion of a predicted transposase gene between *fliD* and *fliS* in *R. intestinalis* L1-82 and the absence of the flagellin gene (*fli*C) from this locus in *E. rectale* A1-86 and *E*. *siraeum* V10Sc8a. The *E. rectale* M104/1 genome also lacks a *fliC* gene at this locus (FP929043.1; ERE_13960–ERE_13910). Neither the separation of the *E*. *rectale* and *R. inulinivorans flgM-csrA* and *flaG-flgN/fliC* gene clusters from each other, nor the absence of flagellin genes from these genomic loci in *E*. *rectale* and *E. siraeum* were due to breaks in the respective draft genome assemblies.

A four-gene motility operon was also present in four of these genomes (Figure 1, panel B). This operon included homologs of *flgF* and *flgG*, two genes which encode structural proteins of the flagellar rod and which were flanked by an MreB-like gene (*mbl*) to the 5′ end, and *flgJ*, a muramidase, to the 3′ end. This operon was absent from the *E. siraeum* genome, because *flgF* and *flgG* were within the largest of the motility loci beside the other genes encoding structural components of the basal body. The *E. rectale* genome included a *flgF-flgG-flgJ* arrangement, but lacked an *mbl* homolog at this locus.

The extent of sequence conservation across these motility loci was examined with Artemis Comparison Tool (ACT) plots. The motility loci of *E. rectale*, *E. eligens* and the three *Roseburia* species were similar. Although the genetic organization of the *E. siraeum* motility loci was comparable to those of the other species studied, it was the most distinct, reflecting the different phylogenetic grouping of this species. The primary sequence of this region was less well conserved, illustrated by the lower level of sequence relatedness visible in Figure S1.

### Isolation, size determination and amino-terminal sequencing of the flagellin proteins of *E. rectale* and *R. inulinivorans*

**Figure 2:**
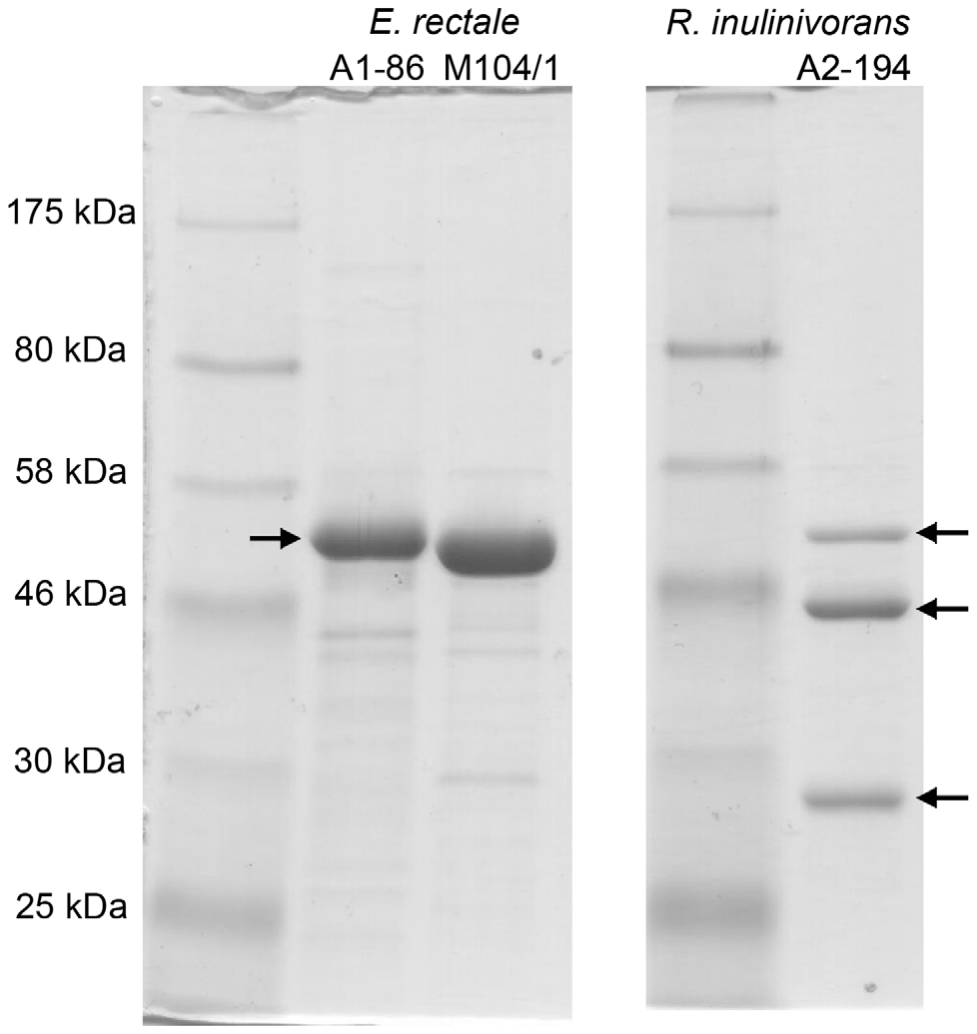
Flagellin proteins from *E. rectale* and *R. inulinivorans* separated on Coomassie stained SDS-PAGE gels. Arrows indicate the proteins for which amino terminal sequence data is available. The broad-range, pre-stained protein marker used (P7708S) was purchased from New England Biolabs.

Separation of the flagellin proteins recovered from *E. rectale* A1-86 and M104/1 by SDS-PAGE revealed a single, major protein band at ∼50 kDa. In contrast, three major protein bands ranging in size from ∼28 kDa to ∼50 kDa were identified in the *R. inulinivorans* A2-194 flagellin preparation (Figure 2). The first ten residues at the amino-terminus of these candidate flagellin protein bands from *E. rectale* A1-86 and *R. inulinivorans* A2-194 (four bands in total) were sequenced and were found to be almost identical (Table S2). These sequences were compared to the translated *fliC* sequences from each genome.

Five *fliC* genes were annotated in the *E. rectale* A1-86 genome and the predicted molecular masses of these flagellin proteins were similar, ranging from ∼47 to ∼53 kDa (Table 1). Five proteins of such similar molecular weights would not have been separated under the SDS-PAGE conditions used here. The first ten residues of four of these predicted flagellin proteins are identical, and matched the chemically determined amino-terminal sequence of the ∼50 kDa protein band exactly. The flagellin protein encoded by the coding DNA sequence (CDS) EUR_28730, is similar in size (∼50.78 kDa), but only four of its amino terminal residues were conserved with respect to the other proteins.

**Table 1:** Summary of the properties of *Eubacterium* and *Roseburia* flagellin proteins and their predicted promoter and ribosome binding site sequences.

Species	No. Flagellins	Locus Tag	Phylogenetic Clade (Fig. S2)	Accession	Size (aa)	Size (kDa)	Sequence of first ten residues	Predicted −35 sequence*	Predicted −10 Sequence*	−35 −10 spacing (bp)	Predicted sigma factor*	−10 to start-codon spacing (bp)	Predicted RBS	RBS-Start codon spacing (bp)
*R. hominis* L1-83	3	RHOM_15820	(a)	AEN98270.1	506	54.48	MRINYNVSAS	taaa	gcgatat	9	28	261	AGGAGA	8
		RHOM_00820	(d)	AEN95291.1	275	30.62	MVVNHNMAAI	taaa	tcgatat	17	28	47	AAGAGG	9
		RHOM_00665	(e)	AEN95260.1	270	28.60	MVVQHNLTAM	taaa	ccgatat	16	28	136	AGGAGG	8
*R. intestinalis* L1	4 (5)	ROSINTL182_05247^†^	(a)	ZP_04742102.2	486	51.90	MRINYNVSAA	taga	ccgatat	15	28	78	AGAAGG	9
		ROSINTL182_08635^†^	(c)	ZP_04745261.1	539	56.13	MVVQHNMSAM	taaa	–	–	–	–	CGGAGG	14
		ROSINTL182_05608	(d)	ZP_04742436.1	275	30.55	MVVNHNMALI	taaa	tcgatat	17	28	47	AAGAGG	9
								tttaca	cataaa	9	43	24		
		ROSINTL182_07256	(e)	ZP_04743973.1	272	29.04	MVVQHNMTAM	taaa	ccgatat	16	28	149	AGGAGG	9
		ROSINTL182_09568	–	ZP_04746122.1	61	6.97	MTLIQNRLEY	taaa	–	–	–	–	–	–
*R. inulinivorans* A2-194	6	ROSEINA2194_00754	(a)	ZP_03752351.1	493	52.52	MRINNNMSAV	taag	acgatat	17	28	34	AGAAGG	10
		ROSEINA2194_01954	(d)	ZP_03753535.1	426	47.24	MQVLAHNLAA	taat	ccgataa	27	28	193	AGGAGA	6
		ROSEINA2194_00384^†^	(e)	ZP_03751985.1	270	28.77	MVVQHNMTAA	taaa	ccgatat	16	28	146	AGGAGG	8
								attaca	aataat	12	43	0		
		ROSEINA2194_00549^†^	(f)	ZP_03752147.1	389	42.06	MVVQHNMQAM	tttaca	aataat	18	43	142	CGGAGG	8
		ROSEINA2194_01473	(f)	ZP_03753062.1	392	42.26	MVVQHNLQAM	–	–	–	–	–	AGGAGG	8
		ROSEINA2194_02155	(f)	ZP_03753734.1	466	49.23	MVVQHNMQAM	tgaa	gcgataa	23	28	375	AGGAGG	8
														
*E. eligens* ATCC27750	3	EUBELI_00422	(c)	YP_002929886	497	52.32	MVVQHNMAAM	taaa	–	–	–	–	CGGAGG	8
		EUBELI_00241^†^	(e)	YP_002929724.1	270	28.93	MVVQHNLSAM	taaa	ccgatat	16	28	93	AGGAGG	8
		EUBELI_00264	(e)	YP_002929747.1	270	29.12	MVVQHNLSAM	ttaa	ccgataa	16	28	92	AGGAGG	8
								taaaca	aataat	13	43	52		
*E. rectale* A1-86	5	EUR_28730	(a)	CBK91820.1	476	50.78	MKINRNMSAV	taaa	tcgatat	17	28	69	AGGAAA	9
		EUR_04790	(f)	CBK89689.1	504	53.41	MVVQHNMQAA	tttcca	cataat	9	43	32	AGGAGG	8
		EUR_14430	(f)	CBK90534.1	480	50.22	MVVQHNMQAA	–	–	–	–	–	TGGAGG	8
		EUR_04300	(f)	CBK89645.1	476	50.10	MVVQHNMQAA	tttcca	cataat	9	43	33	AGGAGG	8
		EUR_14450	(f)	CBK90536.1	455	47.51	MVVQHNMQAA	tttacc	aataat	12	43	22	TGGAGG	8
*E. rectale* M104/1	4	ERE_01930	(a)	CBK92329.1	476	50.77	MKINRNMSAV	taaa	tcgatat	17	28	69	AGGAAA	9
		ERE_14590	(f)	CBK93435.1	458	48.29	MVVQHNMQAM	–	–	–	–	–	AGGAGG	8
		ERE_14720	(f)	CBK93446.1	504	53.41	MVVQHNMQAA	–	–	–	–	–	AGGAGG	8
		ERE_12290^†^	–	CBK93233.1	446	46.82	YRINRAADDA	–	–	–	–	–		
*E. siraeum* V10Sc8a	1	ES1_07000^†^	(b)	CBL33805.1	530	55.81	MVVQHNLNAI	tttaca	tataaa	10	43	258	AGGAGG	17^‡^
								taaa	ccgatat	17	28	192		
														
*E. siraeum* DSM15702	1	EUBSIR_02119^†^	(b)	ZP_02423261.1	539	56.24	MVVQHNLNAI	tttaca	caaaat	11	43	258	AGGAGG	17^‡^
								taaa	ccgatat	17	28	192		
*E. siraeum* 70/3	1	EUS_23890^†^	(b)	CBK97362.1	547	56.97	MVVQHNLNAI	tttaca	tataaa	9	43	258	AGGAGG	17^‡^
								taaa	ccgatat	17	28	192		

* Sequences were compared to the −35 and −10 recognition sequences for *Butyrivibrio fibrisolvens* σ^28^ and σ^43^, which are −35: TAAA (N16–17) −10: MCGATAa and −35: TTtACA (N19) −10: cATAAT respectively. The general bacterial consensus sequences for σ^28^ and σ^43^ are −35: TAAA (N15) −10: CCGATAT and −35: TTGACA (N15) −10: TATAAT respectively. † Predicted start positions were moved on the basis of alignment to amino-terminal sequences of *E. rectale* and *R. inulinivorans* flagellins. ‡ An alternative start codon exists three residues upstream of the predicted start position. Use of this alternative start codon would yield a distance of 8 bp between the predicted RBS and the start-codon.

Four *fliC* genes were annotated in the genome of *E. rectale* M104/1. The estimated sizes of the translated products of CDSs ERE_14590 (∼48 kDa), ERE_14720 (∼53 kDa) and ERE_01930 (∼50 kDa) are consistent with the size of the major protein product at ∼50 kDa on the SDS-PAGE gel. The CDS ERE_12290 is proximally truncated by a break in the draft genome assembly, and was thus selectively excluded from further analyses.

Six *fliC* genes were annotated in the *R. inulinivorans* A2-194 genome. The predicted molecular masses of these candidate flagellin proteins ranged from ∼29 kDa to ∼53 kDa (Table 1). It appears that the translated product of CDS ROSEINA2194_00384 corresponds to the protein product at ∼29 kDa in the SDS-PAGE gel. The products of CDSs ROSEINA2194_00549 and ROSEINA2194_01473 have predicted molecular masses of ∼42 kDa. These may correspond to the protein product migrating at ∼43 kDa on the SDS-PAGE gel. Indeed, the sequence of the flagellin product of CDS ROSEINA2194_00549 corresponds to this protein band, while the product of CDS ROSEINA2194_01473 differs only at residue 7.

Flagellin products of CDSs ROSEINA2194_01954, ROSEINA2194_02155 and ROSEINA2194_00754 have predicted molecular masses of ∼47 ∼49 and ∼50 kDa respectively, and they may be present in the protein band of ∼50 kDa on the SDS-PAGE gel.

### *In silico* flagellin promoter analysis

The nucleotide sequences upstream of the *fliC* genes in each genome of interest were inspected to identify potential promoter sequences and to infer which sigma factors might direct transcription from each promoter (Table 1). Promoters under the direction of either σ^28^ or σ^43^ were identified by comparison to the consensus sequences identified for these promoters in *Butyrivibrio fibrisolvens* [36], and to the bacterial consensus sequences for promoters controlled by these sigma factors. *B. fibrisolvens* promoter sequences were selected as reference sequences for promoter analysis, because on the basis of 16S rRNA gene relatedness, this species is closely related to the *Roseburia* group [22].

The outcomes of this promoter analysis are reported with reference to the clades in the phylogenetic tree based on flagellin proteins, shown in Figure S2. CDSs corresponding to the flagellins in clades A, D and E were under the presumptive control of σ^28^, with the exception of CDSs ROSINTL182_05608 and EUBELI_00264 which were apparently also controlled by σ^43^. Both σ^28^ and σ^43^ consensus sequences were identified for the CDSs encoding the *E. siraeum* flagellin proteins (clade B), but the σ^28^ sequences were closer than the σ^43^ sequences to the predicted start codons of these CDSs. Potential promoters could not be identified for every CDS with a corresponding protein in clade F. The CDSs for which promoters could be identified were mostly under the control of σ^43^.

The inferred σ^28^ and σ^43^ promoters varied considerably in their distance from the predicted CDS start codons, (σ^28^: range, 47–375 bp; mean  = 139 bp. σ^43^: range, 0–258 bp; mean  = 108 bp). The unconventional spacing between the predicted −35 and −10 recognition sequences, and the lack of absolute conservation in the predicted recognition sequences, suggests that if the predicted σ^28^ promoters of ROSEINA2194_01954 and ROSEINA2194_02155 are functional, transcription from these promoters could be suboptimal. This could explain the variable abundance of flagellin proteins in *R. inulinivorans* cultures (see later section). Promoter analysis in *E. rectale* M104/1 was hindered because the regions upstream of the target CDSs were often disrupted by gaps in the draft genome assembly. No potential σ^28^ or σ^43^ promoter sequences were identified upstream of *fliC* CDS EUBELI_00422, ROSINTL182_09568 or ROSINTL182_08635.

### *In silico* and *in vitro* analysis of the pro-inflammatory potential of flagellin proteins from *Eubacterium* and *Roseburia* species

**Figure 3:**
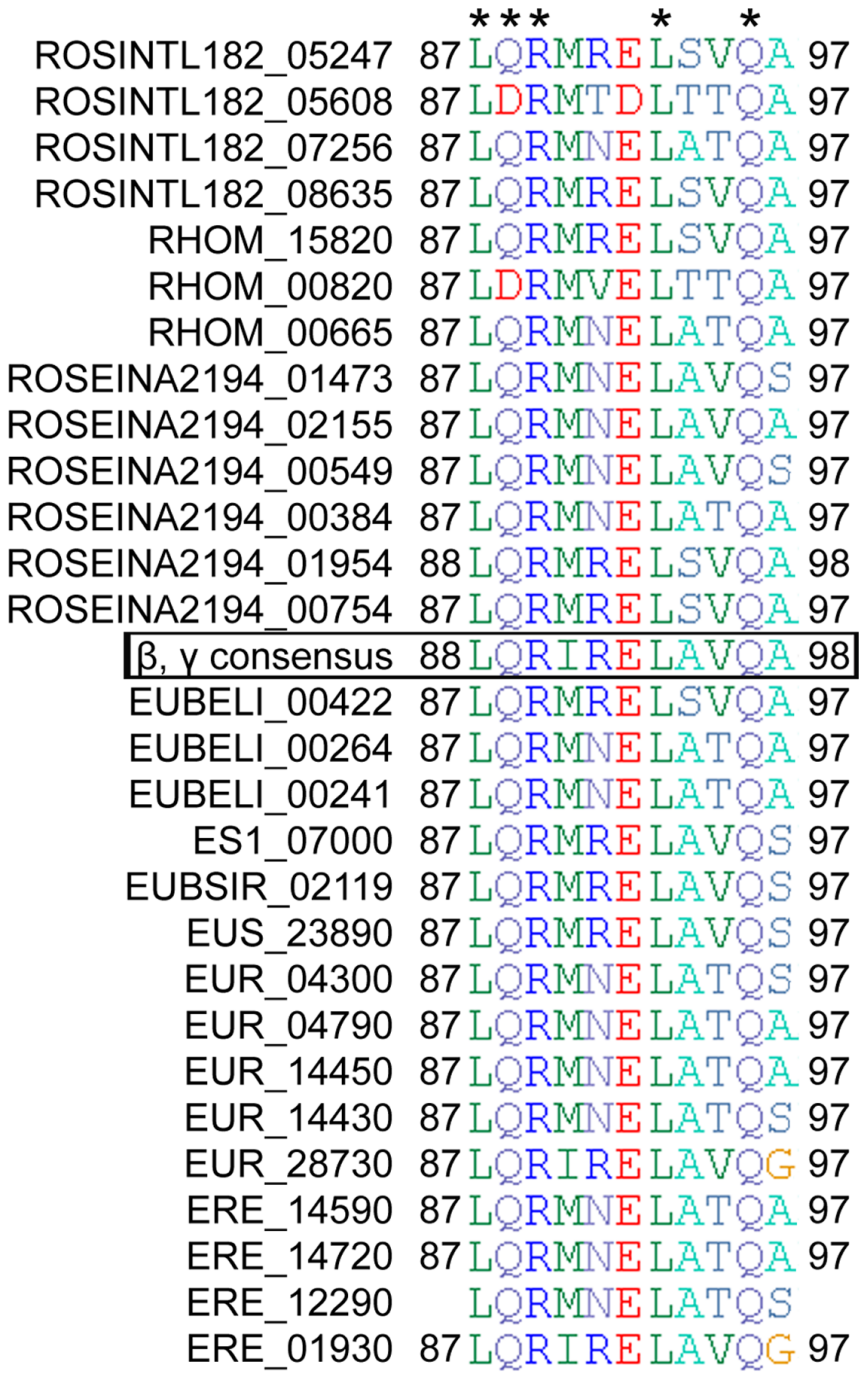
Multiple alignment of the consensus region of the flagellin proteins of β and γ proteobacteria that is recognized via TLR5 with the corresponding regions of predicted flagellin proteins from the *Roseburia* and *Eubacterium* species studied. Residues that are critical for TLR5 recognition are indicated with an asterisk. Alignment was performed with ClustalW in BioEdit. Flagellin proteins from the various species are labelled with a locus tag. A gap in the draft genome assembly meant that positional information could not be included for the sequence fragment of CDS ERE_12290 in this alignment. ROSINTL182 =  *R. intestinalis* L1-82, RHOM  =  *R. hominis* A2-183, ROSEINA2194 =  *R. inulinivorans* A2-194, EUBELI  =  *E. eligens* ATCC27750, ES1  =  *E. siraeum* V10Sc8a, EUBSIR  =  *E. siraeum* DSM15702, EUS  =  *E. siraeum* 70/3, EUR  =  *E. rectale* A1-86, ERE  =  *E. rectale* M104/1.

To predict if the *Eubacterium* and *Roseburia* flagellin proteins were likely to be pro-inflammatory, these proteins were aligned to a consensus sequence (11 residues long) derived from a region of the pro-inflammatory flagellins of the β- and γ- proteobacteria [37], [38]. Residues L87, R89, L93 and Q96 of the *Eubacterium* and *Roseburia* flagellin proteins inspected here were absolutely conserved with respect to the consensus sequence (Figure 3). These residues are critical for TLR5 signalling and flagellin polymerization [37], [38]. Another residue, Q88, that is critical for signalling and polymerisation, is also completely conserved in each of the *Eubacterium* and *Roseburia* sequences with respect to the β- and γ- proteobacteria flagellin consensus sequence, except for the translated products of CDSs ROSINTL182_05608 and RHOM_00820, in which a Q88D substitution is evident. On the basis of their overall similarity to the consensus sequence, these proteins were predicted to have pro-inflammatory properties.

**Figure 4:**
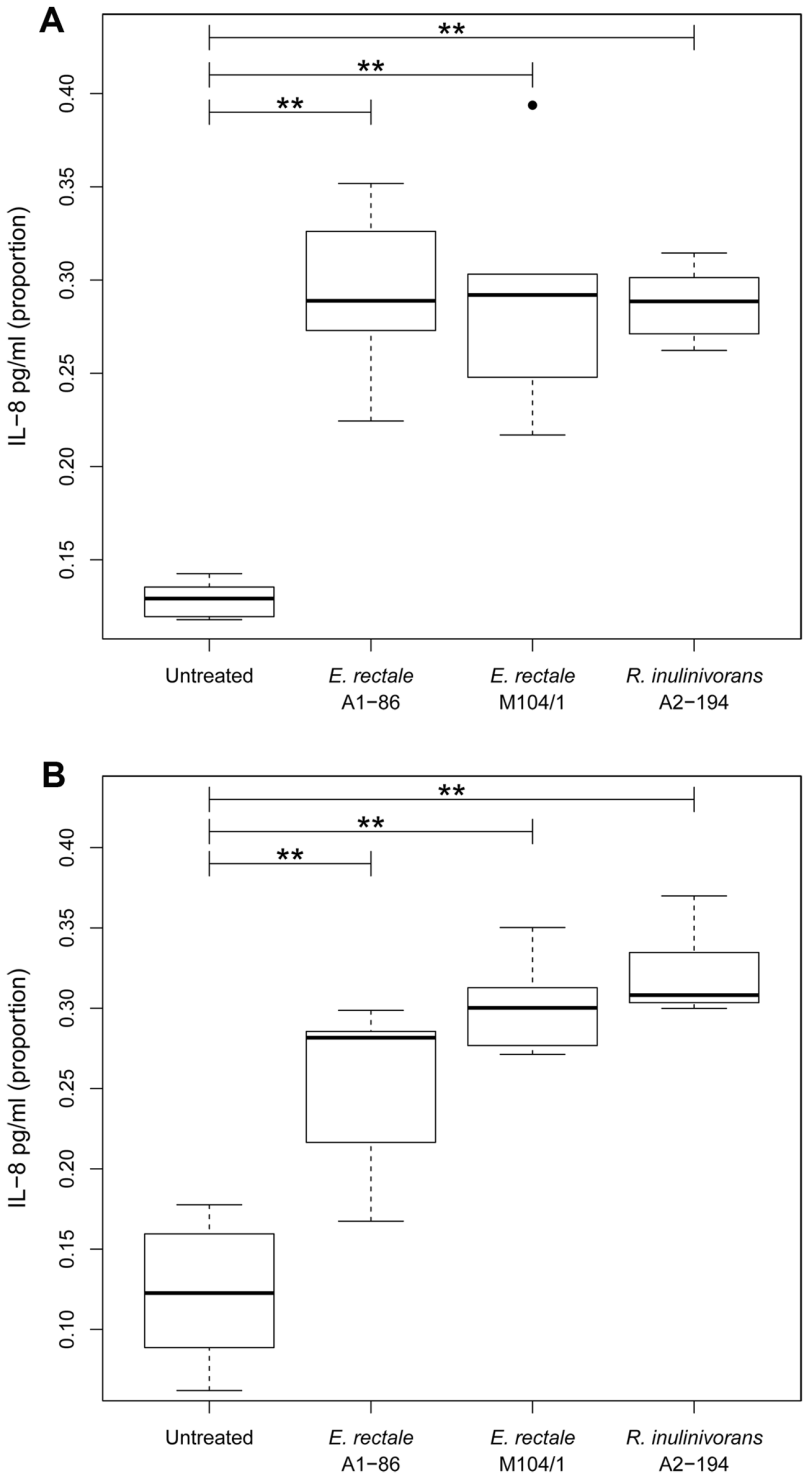
IL-8 secretion from T84 cells (A) and HT-29 cells (B) in response to flagellin preparations from *E. rectale* and *R. inulinivorans.* Concentrations of IL-8 as determined by ELISA were converted to proportions (as described in materials and methods) for statistical analysis. Boxplots show the median value and interquartile range. Outliers are indicated by a black dot. Horizontal bars with the ** symbol indicate that significantly more IL-8 was secreted from the cells treated with flagellin preparations than from the untreated control cells, P-value <0.01, one-tailed Mann-Whitney U test, n = 5 for T84 cells, n = 6 for HT-29 cells.

Two human intestinal epithelial cell lines (IECs), T84 and HT-29, were exposed to the flagellin proteins isolated from *R. inulinivorans* A2-194 and *E. rectale* strains A1-86 and M104/1. Both of these cell lines are suitable for the measurement of IL-8 secretion in response to flagellin preparations, and have been used for this purpose previously [39]. Increased IL-8 secretion by the IECs in response to these flagellin preparations was taken as evidence of a pro-inflammatory response. Significantly more IL-8 was secreted from T84 cells and from HT-29 cells treated with each of the *Eubacterium* and *Roseburia* flagellin preparations than from the untreated control cells (one-tailed Mann-Whitney U test, P≤0.01, n = 5; n = 6 respectively) (Figure 4).

### Identification of selected *Eubacterium* and *Roseburia* species in 27 individual metagenomes

MetaPhlAn [40] was used to determine the relative abundance of 5 of the 6 species of interest in a metagenome database derived from the faecal microbiotas of 27 elderly individuals [41]. The relative abundance of *R. hominis* was not considered using this method because its genome was not included as part of the Integrated Microbial Genomes system, upon which the MetaPhlAn clade-specific marker database was based [40]. Metagenomes EM039 and EM173 were excluded from the MetaPhlAn analysis. These two metagenomes were prepared using alternative sequencing and assembly strategies, which meant that the MetaPhlAn results generated from these two metagenomes were not directly comparable to those from the other 25 metagenomes [41].

According to MetaPhlAn's read-based classification, 23 of the 25 metagenomes harboured at least one of the five species of interest at a relative abundance ≥0.5% (Table S3). Twenty of the 25 metagenomes harbored at least one species of interest at a relative abundance of ≥1%. The relative abundances of each species varied considerably across the metagenomes, and the range of *E. siraeum* relative abundance in particular, was quite large (0.01% (EM191) –31.59% (EM305)). Five of the individuals harbored this species at a relative abundance >3%. Eight people harboured *E. siraeum* at a predicted relative abundance of ≤0.1%.

Significant differences were found in relative abundance for *E. rectale* (Kruskal-Wallis test, H = 10.095, 2 df, P<0.01) and *R. intestinalis* (Kruskal-Wallis test, H = 10.263, 2 df, P<0.01) in the community versus long-stay settings, with significantly higher relative abundances (P<0.05) of these species being recorded in community dwelling individuals, (*E. rectale*, 0.92% community versus 0.045% long-stay; *R. intestinalis*, 0.65% community versus 0.095% long-stay, median values). The relative abundance values of *E. rectale* were also significantly higher for individuals from the rehabilitation setting than from long-stay, (*E. rectale*, 1.075% rehabilitation versus 0.045% long-stay, median values). Relative abundance values of *R. intestinalis* were significantly greater in individuals from the community than in rehabilitation (*R. intestinalis* 0.65% community versus 0.11% rehabilitation, median values). As *E. rectale* and *R. intestinalis* are important butyrate-producing species, these observations are consistent with the findings of a previous study which determined that gene counts for butyrate, acetate and propionate production were significantly greater in the metagenomes representing individuals from the community and rehabilitation settings than from those in long-stay [34]. The relative abundances of *E. eligens*, *E. siraeum* and *R. intestinalis* that were predicted by MetaPhlAn were concordant with the relative abundances of these species that were previously predicted in this cohort by analysis of sequencing reads from the V4 region of the bacterial 16S rRNA gene [21]. It was not possible to deduce the relative abundances of the other target species by this 16S rRNA gene analysis because the V4 region did not offer sufficient resolution at a species level.

The 16S rRNA gene based, strict species abundance values were used however, to test for an association with TNF-α levels in these elderly individuals using Spearman's rank correlation. A significant association of species abundance and TNF-α was confirmed only for *E. siraeum*, rho value  = −0.54, P-value  = 0.007, (P-value  = 0.034 after adjustment for multiple testing), although five species of interest were tested. These results were replicated when the MetaPhlAn-derived relative abundance data rather than the 16S rRNA gene based relative abundance data were used in the analysis. Serum TNF-α levels were lower in individuals that harbored *E. siraeum* at greater than 0.15% (strict species 16S rRNA gene analysis) or 0.25% (MetaPhlAn prediction) relative abundance, depending on the relative abundance measure used (Figure S3).

Recruitment plots of the whole genome sequences of the species of interest aligned to each of the individual metagenomes indicated that the genomes of species present at less than 1% relative abundance were incompletely represented in the metagenomes (data not shown). For some species present at more than 1% relative abundance, discrete genomic regions were apparently not represented in the database. These could represent strain-specific hypervariable sequences, genomic regions that were lost from the non-laboratory strains of these species, or they could represent genomic regions that were excluded from the metagenome assembly. The sequencing coverage for each genome of interest was calculated as a function of metagenome sequencing depth, average target genome size and the predicted relative abundance. The species of interest were often represented at less than 10 fold coverage in these metagenomes (Table S4). This level of genome coverage would probably be insufficient to represent the genomes of interest completely [21], [42], [43].

### Identification of *Eubacterium* and *Roseburia* motility genes in the faecal metagenomes of 27 elderly individuals

The detection of motility CDSs from raw reads was a function of target CDS length and species relative abundance (Figure S4). The number of mapped reads per CDS was normalized to account for sequencing depth differences in each metagenome (see Materials and Methods). The number of raw reads that were mapped to each target CDS increased with both CDS length and the relative abundance of the species of interest in each metagenome. Thus, long CDSs could be detected at lower species relative abundances than short CDSs (Figure S4).

In general, at a species relative abundance of ∼0.1% or greater, ∼10 (Log_10_1) reads (normalized value) were mapped to most of the target genes from each species (Figure S4), and the target DNA sequence was considered as “present” in the sequenced metagenomes. At species relative abundance values greater than or equal to ∼0.4%, more than ∼32 reads (Log_10_1.5) (normalized value) mapped to each target CDS, strongly suggesting that the target DNA sequences were present in the database. In general, homology based methods could identify target genes from assembled metagenomes only when the larger of these species abundance thresholds was exceeded (Table S5). However, motility CDSs were not always detected from raw read databases when a species occurred at a relative abundance ≥0.4%. For example, the species *R. inulinivorans* was estimated at 1.41% relative abundance in EM251 and the corresponding heat-plot suggests that many of the unassembled reads from this metagenome mapped to the target motility CDSs (Figure S4). However, no genes of the *flgB-fliA* motility locus were detected in the assembled metagenome database for this individual by either the homology and annotation or recruitment plot methods (Table S5, Data not shown). Similarly, metagenome EM326 appeared to harbor a complete set of motility genes for *E. eligens*, a species which occurred at 1.54% relative abundance in this metagenome (Figure S4). However, a recruitment plot indicated that few genes at the *flgB-fliA* motility locus of this species were present in the assembled EM326 metagenome (Data not shown).

The heat-plots also show that the genomes of interest were sometimes incompletely represented by the raw unassembled reads. For example, zero or very few reads mapped to the *E. rectale flgB-fliA* motility locus in metagenomes EM148, EM175, EM205 and EM232, even though *E. rectale* was determined to occur at high relative abundances (>0.9%) in these metagenomes. Similarly, target *E. eligens* motility genes were non-uniformly detected in the metagenomes examined, even when this species occurred at high (>1%) relative abundance.

Homology searches and gene context information were used to determine if motility genes of the *flgB-fliA* and *flaG-flgN/fliC* motility loci from the species of interest could be identified from assembled metagenomes. At least some of these *Eubacterium* and *Roseburia* motility genes of interest from the *flgB-fliA* or *flaG-flgN/fliC* motility loci were identified in 23 of the 27 assembled metagenomes (Table S5). *E. siraeum* motility CDSs were identified in 11 of these 23 metagenomes. Motility CDSs from two or more of the target species were detected in 11 of these 23 metagenomes. No single metagenome appeared to harbor complete motility gene sets for all the bacterial species (Table S5).

There was overall correspondence in the detection of *E. siraeum*, *R. intestinalis* and *R. inulinivorans* motility genes from raw and assembled reads (Figure S4, Table S5), though target motility CDSs could be detected at lower species relative abundances when using raw reads compared to when using assembled metagenomes according to the search criteria used. Our inability to detect the motility genes of species that are apparently present in the metagenome database could be a consequence of the incomplete representation of the genome of interest in the metagenome database arising from a non-uniform distribution of sequencing coverage across a target genome, DNA degradation prior to metagenome library sequencing, or the loss or divergence of these regions in intestinal strains of these species.

To evaluate the overall abundance of cell motility genes in these assembled metagenomes, the number of “cell motility” clusters of orthologous groups (COG) (category N) associated with each metagenome was investigated (Table S6). This category includes 96 individual COGs which specify functions involved in flagellum biogenesis, chemotaxis and pilus assembly (Table S7). [44]. The number of “cell motility” COGs represented by each assembled metagenome varied considerably, ranging from 2 COGs (EM227) to 19 COGs (EM283). Accordingly, the proportion of COGs assigned to this functional category varied across the metagenomes, and ranged from 0.13% (EM227) to 0.87% (EM205, EM326) of total COGs assigned to any category per metagenome. Thus, the function of “cell motility” was not abundantly encoded in any of these assembled metagenomes.

### Identification of *Eubacterium, Roseburia* flagellin genes and proteins in the assembled faecal metagenomes of 27 elderly individuals

The presence of flagellin proteins in each of the 27 metagenomes was evaluated with fragment recruitment plots (Figure S5) and also by BLAST searches. The recruitment plots revealed that the flagellin proteins of the species of interest were present in 8 of the 27 metagenomes. Two of the four full-length *R. intestinalis* flagellins (ROSINTL182_05608 and ROSINTL182_07256) were only represented in metagenome EM268. Of the six *R. inulinivorans* flagellins, only the product of *fliC* CDS ROSEINA2194_00754 was identified, and was represented in two metagenomes, EM268 and EM175. Partial matches to *R. inulinivorans* flagellin proteins encoded by ROSEINA2194_00754 and ROSEINA2194_01954 were identified in metagenome EM173.

The protein product of *E. rectale* CDS ERE_01930 was the only *E. rectale* flagellin represented in 5 metagenomes (EM039, EM205, EM251, EM268, EM219). The protein encoded by CDS ERE_01930 is 99% similar to EUR_28730, and would explain why a non-identical, but highly similar homolog of EUR_28730 occurs in every metagenome that also encodes an identical match to ERE_01930. The *E. siraeum* 70/3 flagellin protein encoded by CDS EUS_23890 was present only in metagenome EM039. Homologs of this flagellin which are 74% and 88% identical to EUS_23890 respectively from other *E. siraeum* strains were not identified in any of the metagenomes examined. However, a protein similar to the *E. siraeum* flagellin encoded by CDS ES1_07000 was identified in metagenome EM176. *E. eligens* flagellin proteins were not identified in any of the metagenomes by this method. Recruitment plots could not be constructed for metagenomes EM208, EM227, EM238 or EM275 because no informative alignment data were returned by the analysis, indicating that these flagellin proteins were not represented in the recruitment plots above the thresholds used (which is consistent with results presented above). When a flagellin protein of interest was detected at 100% similarity by the recruitment plot method, the other flagellin proteins of this species were not also detected. Filtered tBLASTn searches (≥90% minimum identity, E-value ≤1.0×10^−8^, ≥250 residues long) suggested that *Eubacterium* and *Roseburia* flagellins were represented in 8 metagenomes (EM039, EM175, EM204, EM205, EM209, EM219, EM351 and EM268). EM268 harbored sequences which aligned to five flagellins (ROSINTL182_07256, ROSINTL182_05608, ROSINTL182_05247, ROSEINA2194_00754 and one sequence that aligned to both ERE_01930 and EUR_28730). The equivalent *E. rectale* flagellin homologs from two different strains (ERE_01930 and EUR_28730) aligned to sequences in 5 metagenomes, (EM039, EM205, EM219, EM251, EM268). The *E. siraeum* flagellin EUS_23890 aligned only to EM039. Flagellin proteins ROSINTL182_05247, ROSEINA2194_00384 and ROSEINA2194_00754 aligned to metagenomes EM209, EM204 and EM175 respectively. Flagellins ROSEINA2194_00754, ROSINTL182_05608, ROSINTL182_07256 and ROSINTL182_05247 also aligned to EM268 under the thresholds used.

Sequences that could be assigned to COG1344, which represents “flagellin and related hook-associated proteins”, were present in 23 of the 27 assembled metagenomes (Table S6). Because this analysis was performed on assembled metagenomes, it only indicates the presence or absence of the target COGs in the metagenome databases, and does not provide the overall abundance of particular COGs. Metagenomes EM148, EM204, EM227 and EM308 did not harbor any sequences that could be assigned to this COG category. This automated functional analysis therefore suggests that “flagellin and related hook-associated proteins” are variably represented in these metagenome databases.

In the gut, the genes encoding flagellin are unevenly distributed among the various lineages of intestinal bacteria. When flagellin proteins from either *Bacillus subtilis* (NP_391416.1) or *Salmonella enterica* subsp. *enterica* serovar *Typhimurium* (NP_460912.1) were used as BLASTp queries to search a collection of publically available human gut bacterial genomes [16] for flagellin orthologs, only species of the genera *Anaerobaculum*, *Anaerotruncu*s, *Butyrivibrio*, *Citrobacte*r, Clostridium*, *Enterobacter*, *Escherichia*, Eubacterium*, *Helicobacter*, *Listeria*, *Roseburia, Providencia*, yielded positive matches according to the threshold values used to define orthologs (at least 30% identity over at least 80% of the query length). (Not all target species of the genera marked with an asterisk harbored a flagellin ortholog).

## Discussion

Due to their production of flagella, the motile *Eubacterium* and *Roseburia* species have considerable immunostimulatory potential. While motility may be a colonization factor for enteric *Roseburia* species [45], [46], the expression of flagellin proteins that are recognized by human TLR5 nevertheless confers a pro-inflammatory capacity upon these species [29]. By *in silico* analysis, the flagellin proteins of the *Eubacterium* and *Roseburia* species studied here were all predicted to be pro-inflammatory, and this pro-inflammatory capacity was experimentally supported for the flagellin proteins isolated from strains of *E. rectale* and *R. inulinivorans*. These findings are consistent with those of previous studies, which demonstrated that whole cells and conditioned media from species of this phylogenetic cluster could activate NF-κB or expression from an NF-κB reporter construct [13], [27]. Although NF-κB is often activated in response to pathogenic infections, its activation is not necessarily undesirable, and the pro-inflammatory flagellin proteins characterized here could contribute favourably to gut health by promoting intestinal epithelial homeostasis and by preventing cell-death and disease [2], [47], [48].

The flagellum biogenesis pathway in bacteria is hierarchically regulated. The basal-body and hook are synthesized before the filament is assembled [49], [50]. Specific intermediate stages in the flagellum assembly pathway serve as checkpoints which coordinate the expression of flagellum biogenesis genes [50]. Thus, the arrangement of genes in operons and/or transcriptional units which reflect the order of their temporal expression is a common feature of bacterial flagellar systems which contributes to the efficient regulation of flagellum biogenesis [51], [52]. The genetic organization of motility genes in the *Eubacterium* and *Roseburia* genomes was consistent with that found in other motile species of the phylum *Firmicutes* [10]. Gene order is known to become less conserved with increasing genetic distance between species [53]. Consistent with this, the genetic organization of the major motility loci were very similar among the *Lachnospiraceae* genomes investigated, but the *E. siraeum* motility locus was quite different to the others at a sequence level and with respect to gene content, reflecting its phylogenetic positioning in *Ruminococcaceae*.

The *Eubacterium* and *Roseburia* motility genes were found at various loci throughout each genome, as is the case with several *Clostridium* and *Bacillus* species. The genes in the largest of the *Eubacterium* and *Roseburia* motility loci encode the structural and regulatory components of the basal-body and hook. These are expected to be transcribed early in the flagellum biogenesis pathway to anchor the flagellum in the cell membrane. The organization of the genes for the structural, chaperone and regulatory functions involved in flagellar filament formation at another motility locus (*flgM-flgN/fliC*) may enable the efficient regulation and timely expression of these genes. In support of this hypothesis, a similar gene arrangement occurs in a number of other bacterial lineages [54].

In four of the genomes studied, two genes encoding structural rod proteins, *flgF* and *flgG*, which transmit torque from the motor to the hook and filament were found in a separate four gene operon, with *mbl* and *flgJ* located immediately up- and down- stream of the *flgF- flgG* gene pair respectively. The *mbl* gene encodes an MreB-like protein which has a role in determining cell morphology and polarity [55]. The FlgJ protein is a rod-specific muramidase with peptidoglycan hydrolyzing ability that is exploited during the construction of transmural flagellar structures [56]. In some *Firmicutes* species [10] including *E. siraeum* V10Sc8a, *flgF* and *flgG* are found in an operon with the genes for other basal body and rod proteins [10]. However, the *mbl-flgF-flgG-flgJ* genetic arrangement described here is also found in the genomes of several closely related *Butyrivibrio* and *Clostridium* species from *Lachnospiraceae* and *Clostridiaceae* families and in *Alkaliphilus oremlandii* (also family *Clostridiaceae*) and *Abiotrophia defectiva* (class *Bacilli*). The *E. rectale* FlgF and FlgG proteins are 54% (154/282 aa) and 50% (141/282 aa) similar to *Bacillus subtilis* subsp. *subtilis* FlhO (CAB05950.1) and FlhP (CAB05941.1) respectively, suggesting that these proteins are homologous. The *mbl-flhO-flhP* gene arrangement occurs in *Bacillus, Geobacillus* and *Oceanobacillus* species. The functional and evolutionary significance of the *mbl–flgJ* genetic arrangement is presently unknown.

Flagellin expression is known to occur at higher levels in *R. inulinivorans* A2-194 when it is grown on starch rather than on glucose, inulin or fructan substrates [46]. This nutritional control of motility gene expression implies that pleiotropic global regulators may direct motility gene transcription or translation in *Roseburia* species. Under nutrient rich conditions, CodY represses flagellin expression in *B. subtilis* [57]. A *codY* homolog was identified immediately upstream of the *flgB-fliA* motility locus in the *E. rectale*, *E. eligens*, *R. hominis* and *R. intestinalis* genomes examined. In *R. inulinivorans*, the CDS encoding the predicted *codY* homolog (ROSEINA2194_0938) is apparently fused to the 3′ end of a CDS encoding a protein with DNA topoisomerase I function. CsrA, a global regulator that inhibits flagellin gene expression in *B. subtilis* [58], but which is necessary for motility and flagellum biosynthesis in *E. coli* [59] was also found at the *flgM-flgN/fliC* motility locus of all genomes examined. In other species, the activities of CodY and CsrA can be modulated by changes in intracellular guanosine tetraphosphate (ppGpp), guanosine nucleoside triphosphate (GTP) or branched chain amino-acid pools [57], [60]. Unfavourable environmental conditions such as nutrient limitation, induce a stringent response in some bacteria which leads to either motility gene expression or repression by altering intracellular concentrations of these effector molecules [60]. Further experiments would be required to determine which, if any of these effector molecules, modulate motility gene transcription via CodY or CsrA in motile *Eubacterium* and *Roseburia* species during growth on different carbohydrate substrates.

*In silico* analysis of promoter consensus sequences suggested that the *fliC* genes in the *Eubacterium* and *Roseburia* genomes of interest were mostly under the control of σ^28^, although some σ^43^ dependent promoters were also identified. In *B. fibrisolvens,* transcription of one *fliC* gene is driven from two different promoters, yielding two transcripts with alternative transcription start-sites [36]. For the *Eubacterium* and *Roseburia fliC* genes with potentially more than one promoter, it is not yet clear if transcription proceeds from both. The presence of two promoters for a single *fliC* gene, one of which is under the presumptive control of a housekeeping sigma factor, suggests that there may be a requirement for constitutive *fliC* transcription at a basal level in these species. It also suggests that post-transcriptional or post-translational control mechanisms, such as those that have been described for other motile species [54], [58], [61] might additionally regulate flagellin expression in these species.

The motile *Eubacterium* and *Roseburia* species bear subterminal flagella [25], [62] and the annotation of several flagellin proteins in the genomes of these *Eubacterium* and *Roseburia* species suggests that these bacteria might produce complex flagella in which the filament is composed of several different flagellin proteins. This inference is supported by the recovery of at least three flagellin proteins from *R. inulinivorans* cultures. It is possible that *E. rectale* also produces complex flagella, but the sizes and amino-terminal sequences of its flagellins were insufficiently unique to determine which of its flagellins were expressed. In contrast, only one flagellin gene was annotated in each of the genomes of three *E. siraeum* strains, so this species presumably produces flagella composed of a single flagellin protein. Gene gain by duplication or horizontal gene transfer could explain the occurrence of multiple genes encoding flagellin in the genomes of these species of interest.

We attempted to identify motility CDSs of specific motile, enteric *Eubacterium* and *Roseburia* species from the raw read and assembled metagenome datasets generated by the ELDERMET project [41]. These databases were selected for analysis because the average N50 size of the assembled metagenomes was large, ∼24 kb. (The average N50 for individuals from different community settings varied considerably from ∼16.4 kb (community) to ∼339.5 kb (long-stay), depending on the diversity of the intestinal microbiota present [41]). This average contig N50 value exceeded the N50 values reported for the assembled metagenomes of another intestinal metagenome database [16]. Due to these fundamental differences in metagenome structure, target gene detection in other metagenome databases was not considered.

Our heat-plots showed that the identification of motility CDSs from databases of unassembled reads was a function of both target gene length, gene context and target species relative abundance. Longer CDSs would, therefore, be detected at lower species relative abundances than shorter CDSs (Figure S4–A). At species relative abundances of ∼0.1%, unassembled reads mapped non-uniformly to the target motility loci (Figure S4), implying an uneven depth of sequencing coverage of the target genome at this level of species relative abundance.

The proportion of raw sequencing reads returned for any given genome in a metagenome database corresponds to the relative abundance of the target species in the sampled environment, and to its genome size. Abundant species are therefore expected to have greater genome coverage than less abundant species. Species with larger genomes are expected to have less genome coverage than species with smaller genomes, assuming that their relative abundances in a specific metagenome, are the same. For example, in metagenome EM175, *E. rectale* occurs at 2.06% relative abundance, and has a predicted coverage of 28.12 fold. In the same metagenome, *R. inulinivorans* is more abundant (2.23%), but has less genome coverage (26.28 fold) due to its larger genome size.

Notwithstanding the effect of genome size on sequencing coverage, the heat-plots (Figure S4) show that target genes were more readily detected in metagenomes when these species were present at a high relative abundance. This was attributed to the greater depth of sequencing coverage of these high abundance genomes. Deeper genome coverage would therefore be expected to improve gene detection in low abundance species, or in species with very large genomes. Nevertheless, the depth of sequencing used in the preparation of these metagenomes is comparable to those used in another intestinal metagenomics project [16].

In metagenomes that were thought to include *E. rectale* at high (≥1%) species relative abundances, the apparent absence of the *E. rectale flgB-fliA* motility locus was unexpected. Technical issues, such as DNA degradation or a DNA sequence composition which was refractory to sequencing might explain the lower than expected coverage of this region in databases of raw reads. Alternatively, the divergence or loss of this region in enteric *E. rectale* strains would also preclude the detection of these target motility genes by comparison to the reference genome of a laboratory strain.

We suspect that incomplete sequence coverage of the target bacterial genomes also imposed a limitation on our ability to identify specific genes or pathways from the assembled metagenomes. The assembly status of the query genome and the metagenome database may also influence the outcome, because more fractured assemblies yield shorter alignments. Thus, even at the large sequencing depths (3317 to 4798 Mb) and metagenome contig lengths (2050 bp ≤N50 ≤64999 bp) used here [41], these metagenomes appear to incompletely capture the total functional diversity encoded at a species level in these faecal microbial communities.

Consistent with earlier studies [17], our recruitment plot and COG analyses suggest that genes encoding cell motility functions occur at variable and low abundances in the human gut microbiome. Indeed, orthologs of flagellin proteins were identified in the genomes of only a subset of human gut bacteria. Poor coverage of low abundance genomes is a known current limitation of metagenomics [63] and gene finding from assembled, but fragmented sequences is a recognized challenge for pathway reconstruction from metagenomes [64]. Our attempt to identify genes involved in bacterial motility from specific high-abundance target species from databases of raw reads and assembled metagenomes, highlights the need for a greater depth and evenness of sequencing or improved metagenome assembly from short reads to improve gene detection and pathway reconstruction.

In summary, we have demonstrated the pro-inflammatory nature of the flagellins of some of the most abundant motile commensal bacteria in the human GI tract *in vitro* and we have investigated the potential regulation of these genes by *in silico* means. We also highlight the need for greater depth and evenness of sequencing in the preparation of metagenome databases to ensure that the genetic functionality encoded by an ecosystem is fully captured at species level.

## Materials and Methods

### Strains and genomes studied

Three *Eubacterium* species (*E. eligens, E. rectale* and *E. siraeum*) and three *Roseburia* species (*R. hominis, R. inulinivorans* and *R. intestinalis*) were the focus of this study. The specific strains studied are mentioned in Table S8. A summary of the genome assembly statistics for each genome studied is also provided in Table S8. The genomes of *E. rectale* A1-86, *E. rectale* M104/1, *R. intestinalis* L1-82, and *E. siraeum* 70/3 were sequenced at the Sanger Institute as part of the MetaHit project, http://www.sanger.ac.uk/pathogens/metahit/.

### Culture conditions

The three strains (*E. rectale* A1-86, *E. rectale* M104/1 and *R. inulinivorans* A2-194) were previously isolated from human faecal samples [65], [66]. The growth medium used was anaerobic M2GSC, prepared as in reference [67]. This medium was divided into 7.5 ml aliquots in Hungate tubes, sealed with butyl rubber septa (Bellco Glass) or 500 ml aliquots in 1 litre laboratory bottles (Duran Group), with specially modified airtight caps. All cultures were inoculated using the anaerobic methods described by Bryant, 1972 [68] and incubated anaerobically at 37°C without agitation. In brief, carbon dioxide gas was diffused through the growth medium before dispensing and sealing in an airtight vessel. Carbon dioxide was pumped into the overnight cultures and into the fresh medium to maintain the anaerobic conditions during inoculation.

In order to obtain sufficient quantities of flagellin protein, large batches of bacterial culture were grown anaerobically: Two overnight 7.5 ml cultures of M2GSC broths were used to inoculate each single anaerobic bottle containing 500 ml M2GSC. Duplicate bottles were prepared for each strain. These subcultures were incubated for 16–18 hours before harvesting the flagellin proteins using methods outlined previously [39].

### SDS-PAGE, staining, quantification and amino-terminal sequencing of flagellin proteins

Flagellin proteins were electrophoresed on 10% SDS-PAGE gels and were visualized by staining with Coomassie blue stain followed by destaining with “destain solution” (methanol: acetic acid: water, 454: 92: 454).

Proteins separated by electrophoresis were transferred to Immobilon membrane for amino-terminal sequencing. Transfer of proteins was performed at 40 mA for 50 mins in transfer buffer (1× CAPS (Sigma, Catalog No., C2632); 100 ml methanol; 800 ml water). The membrane was stained and destained post-transfer to visualize the proteins. The protein bands of interest were excised from the membrane and the first ten residues of each protein band were amino-terminally sequenced by AltaBioscience, Birmingham, UK.

Proteins were quantified using the BCA protein assay (ThermoScientific Pierce Catalog No., 23225) according to the microplate procedure outlined by the manufacturer.

### Stimulation of intestinal epithelial cells and IL-8 ELISA

HT-29 (ATCC HTB-38) and T84 (ATCC CCL-248) cells were routinely cultured in Dulbecco's Modified Eagle Medium (DMEM) (Sigma Catalog No., D6429) supplemented with 10% foetal bovine serum (Sigma Catalog No., F9665) and 1% penicillin/streptomycin antibiotics (Sigma Catalog No., P4333) stock concentrations: 10,000 U penicillin and 10 mg streptomycin/ml) and were incubated at 37°C in a 5% CO_2_ atmosphere. IECs were seeded at a density of 2×10^4^ cells/well of a sterile 96 well plate. After seeding, IECs were allowed to adhere overnight before flagellin treatment.

Flagellin proteins were added to each well to a final concentration of 0.1 µg/well. Flagellin suspensions of the desired concentration were prepared in DMEM. Exposure of the IECs to flagellin proteins took place for 12 hours. Supernatants were subsequently recovered. The interleukin-8 (IL-8) concentration in these supernatants was measured with the IL-8 ELISA Duo kit (R&D systems) according to the manufacturer's instructions. Experimental replicates were performed on different days. The same concentration of flagellin was used as a stimulant in each independent experiment. For statistical analysis, the raw IL-8 values were converted to proportions by dividing the IL-8 concentration for each treatment in a single experiment by the sum of the IL-8 concentrations for all of the treatments from the same experiment. A one-tailed Mann-Whitney U test was performed on the transformed values.

TNF-α levels in blood samples were determined previously using microplates from Meso Scale Diagnostics [41]. Associations between species relative abundance and TNF-α levels were assessed using the Spearman correlation coefficient.

### Genome annotation and improvement, comparative genomics, metagenome assembly

Draft and complete genome sequences were downloaded from the nucleotide database on the National Center for Biotechnology Information website (Table S8). Several of these genomes had previously been annotated by automated procedures. These auto-annotations of motility genes at the major motility loci in the *E. rectale* A1-86 and *R. inulinivorans* A2-194 genomes were inspected. The motility gene arrangements in the other genomes of interest, specifically *E. eligens*, *E. siraeum*, *R. hominis* and *R. intestinalis* (Table S8), were examined with respect to the major motility loci of the *E. rectale* and *R. inulinivorans* genomes. Additional open reading frames that were not previously identified in the auto-annotation of these draft genomes were inferred on the basis of their genetic neighborhood and BLASTp similarity to characterized homologs. The CDSs that represented fragments of genes that apparently included frameshift mutations were merged. Start positions of genes encoding flagellin proteins were adjusted to correspond to the amino-terminal sequence derived for the flagellin proteins that were recovered from *E. rectale* and *R. inulinivorans*.

Assembled metagenomes representing the intestinal microbiomes of 27 elderly Irish individuals from one of three community settings (community, rehabilitation and long-stay) were generated previously [41] and each included on average 4.6 Gb of sequence information. The MG-RAST accession numbers for each of these metagenomes are included in Table S6. Twenty-five of these metagenomes were constructed from libraries of 91 bp paired-end Illumina reads with an insert size of 350 bp. Two of these metagenomes (EM039 and EM173) were assembled using two different types of sequencing technologies, specifically paired-end Illumina reads that were 101 bp in length with a 500 bp insert size in combination with 551,726 and 665,164 454 Titanium sequencing reads for EM039 and EM173 respectively.

### Analyses of presence or absence, relative abundance and extent of genome coverage of *Eubacterium* and *Roseburia* species of interest in metagenomes

MetaPhlAn 1.6.0 [40] was used to infer the relative abundances of the target species in the 27 metagenomes. The “MetaPhlAn script” and the “BowTie2 database of the MetaPhlAn markers” were downloaded from http://huttenhower.sph.harvard.edu/metaphlan. Unfiltered paired-end reads were combined in a FASTQ file which was converted to FASTA format using FASTQ-to-FASTA (FASTX-Toolkit: http://hannonlab.cshl.edu/fastx_toolkit/commandline.html). The output file was subjected to MetaPhlAn analysis using default parameters.

The differences in species relative abundance across the three community settings were investigated by non-parametric analysis methods. A Kruskal Wallace test was performed on the relative abundance values predicted by MetaPhlAn for each species across the three community settings. A Tukey test was performed on the Kruskal Wallace output to determine significant differences in the relative abundances of specific species across the three community groups.

The estimated coverage of each target genome in each of the metagenomes was calculated as a function of the metagenome size, the average size of the target species' genomes and the MetaPhlAn-predicted relative abundance of the species of interest according to the following formula: ((Metagenome size (Mb) × Rel. Abundance (%))/(Target genome size (average) (Mb)) [63]. Average genome sizes were calculated from all genomes sequences available for each species.

### Identification and annotation of motility proteins of *Eubacterium* and *Roseburia* species in metagenomes

Two approaches, based on either raw sequencing reads or reads assembled into contigs, were adopted for the identification of motility genes from the target species of interest in the ELDERMET metagenomes. Bowtie 2 [69] was used with default settings (end-to-end read alignment, –sensitive -D 15 –R 2 –N 0 –L 22 –i S,1,1.25) to map raw sequencing reads from each metagenome to the *Eubacterium* and *Roseburia* ORFs and CDSs of interest. The number of mapped reads was normalized according to the following calculation: (No. mapped reads) × (Mean sequencing depth/Sequencing depth per metagenome). The mean sequencing depth was taken as 4.79×10^9^ bases per metagenome. The total sequencing depth for each metagenome was reported as part of the supporting information accompanying an earlier publication [41].

Heat plots were created with an edited “Heatplot” function as part of the Made4 package [70] for R. These plots were based on the normalized number of mapped reads per gene per metagenome, the MetaPhlAn [40] derived species relative abundance values and target CDS lengths (bp). For metagenomes EM039 and EM173, species relative abundance values were inferred by calculating the relative abundance value that was mid-way between the MetaPhlAn predicted relative abundance values for the species of interest in the metagenomes that occurred immediately adjacent to EM039 and EM173 after all the metagenomes were ranked in order of increasing total number of normalized mapped sequencing reads. Target CDSs were considered as present at a minimum threshold of ∼10 normalized reads mapped per gene (Log _10_1).

A selection of 177 *Eubacterium* and *Roseburia* motility proteins (excluding genes encoding flagellin proteins) which represented the *flgB-fliA* and *flgM-flgN/fliC* motility loci of eight different species (*E. cellulosolvens, E. eligens, E. rectale, E. siraeum, E. yurii* subsp. *margaretiae, R. hominis, R. intestinalis, R. inulinivorans*) were used as tBLASTn queries to search the database of assembled metagenomes for contigs which likely harbored motility genes from the species of interest. The genes encoding flagellins were excluded from this analysis because flagellin domain sequences are often conserved across species [71]. This conservation of amino-acid sequence was expected to yield non-specific BLAST matches. Furthermore, the genes encoding flagellin proteins were often dispersed throughout the genomes, so detection of a flagellin would not always lead to the target *flgB-fliA* or *flgM-flgN/fliC* operon. The metagenome contigs that yielded alignments which were ≥90% identical to the query proteins were retrieved from the database. These contigs were viewed and all potential ORFs were called using Artemis [72]. These ORFs were annotated on the basis of BLASTp homology to proteins in the non-redundant protein database (NR) available from NCBI, and also by a general inspection of their genetic neighborhood. The motility genes of a target species were considered to be present in a target metagenome if the best BLASTp hits for at least half of the motility CDSs on each contig occurred with identity ≥90% to homologs from only one of the target species.

### COG category analysis

The 27 assembled metagenomes [41] are publically available on the MG-RAST website [73]. COG classifications were determined via MG-RAST for each metagenome using default parameters (≥60% identity, ≥15 aa alignment length, E-value ≤1×10^–5^). Data were viewed in tabular output format and were filtered at “level 2” to limit results to “cell motility” COGs. The proportion of COGs assigned to this category was expressed as a percentage of total COGs (total number of COGs returned before filtering).

### BLASTp analysis of publically available human gut bacteria genomes

Flagellin protein sequences from *Bacillus subtilis* subsp. *subtilis* 168 (NP_391416.1) and *Salmonella enterica* subsp. *enterica* serovar *Typhimurium* LT2 (NP_460912.1) were used to query the genomes from a list of 194 publically available human gut bacteria genomes (Supporting Information Table 5 in reference [16]) that were available in the NCBI BLAST database (April 2013). A genome was considered to contain a flagellin ortholog if a BLASTp hit to either of the query sequences occurred with at least 30% identity over at least 80% of the query length.

### Generation of recruitment plots

Recruitment plots were constructed using PROmer 3.07 [74] to align the query sequences to the database of assembled metagenomes. Query sequences were typically complete or draft genome sequences, genomic fragments representing a motility locus of interest or a multi-fasta file representing genes of interest. The PROmer delta output file was filtered using mummerplot 3.5 (part of the MUMmer package) [75]. The plots were generated with a range of 80–100% similarity represented on the Y axis.

### Comparative genomics

Nucleotide and amino-acid alignments were performed with MUSCLE [76] or ClustalW in BioEdit. Artemis Comparison Tool was used to view the conservation and arrangement of large genome segments across species [77]. The comparison files were generated in tabular format using tBLASTx [78]. A minimum identity threshold of 30% was imposed on the alignments for visualization purposes.

### Phylogenetic analysis

Phylogenies constructed from protein sequences were first aligned using MUSCLE [76]. A rooted flagellin protein phylogenetic tree was constructed using PHyML 3.0 [79] with the LG substitution matrix. Modelgenerator [80] was used to choose the most appropriate substitution model. Alignment columns that included gaps were removed before constructing the maximum likelihood tree.

### Promoter sequence analysis

The nucleotide sequences upstream of the genes encoding flagellin proteins were inspected to identify potential sigma factor consensus sequences and ribosome binding sites (RBS). The promoter sequences of the housekeeping sigma (σ^43^) factor (−35: TTtACA, −10: cATAAT) and the flagellar (σ^28^) sigma factor (−35: TAAA −10: MCGATAa) of *Butyrivibrio fibrisolvens* (another motile species of *Clostridium* cluster XIVa) were used as reference sequences [36]. Ribosome binding sites were expected to occur within 20 bp of the predicted start-codon [81], and to conform to the sequence AGGAGG.

## Supporting Information

Figure S1 **ACT alignments of** ***flgB-fliA*** **(top) and** ***flgM-flgN/fliC*** **(bottom) motility loci.** Locus tags indicate which genomic region is represented. A minimum threshold of 30% identity was imposed on the alignments. Alignments involving *E. rectale* and *R. inulinivorans flgM-csrA* and *flaG-flgN/fliC* are on bottom left and right respectively.
(TIF)

Figure S2 **Phylogenetic tree of flagellin proteins.** The flagellin tree was constructed from flagellin protein sequences using PHYML with model LG. Numbers at each node are bootstrap values. Locus tags were used to label flagellin proteins. Strongly supported clades (bootstrap ≥55) are surrounded by coloured boxes and are labelled with a letter A–F. ROSINTL182 =  *R. intestinalis* L1-82, RHOM  =  *R*. *hominis* A2-183, ROSEINA2194 =  *R. inulinivorans* A2-194, EUBELI  =  *E*. *eligens* ATCC27750, ES1  =  *E. siraeum* V10Sc8a, EUBSIR  =  *E. siraeum* DSM15702, EUS  =  *E. siraeum* 70/3, EUR  =  *E. rectale* A1-86, ERE  =  *E. rectale* M104/1.
(TIF)

Figure S3 **Association between** ***E. siraeum*** **relative abundance and serum TNF-α concentration.** Boxplot showing median serum TNF-α concentration which is greater in individuals that harbor *E. siraeum* at <0.15% relative abundance (n = 14), than in individuals that harbor this organism at >0.15% relative abundance (n = 10). Boxplots show the median and interquartile range. Outliers are indicated by o symbols. Significance was assessed using the Spearman correlation coefficient.
(TIF)

Figure S4 **Heat-plots showing the relationship between the normalized number of reads mapped to target motility CDSs as a function of CDS length and target species relative abundance.** Heat-plots labelled “A” show that the normalized number of reads that mapped to each target gene increases with increasing CDS length and species relative abundance. Heat-plots labelled “B” show that the normalized number of reads that mapped to target CDSs varied depending on gene context. For each species, heat-plots A and B present the same data, but differ due to alternative arrangements of the CDSs on the X axis. In heat-plots labelled “A”, CDSs are arranged according to increasing length, while in heat-plots labelled “B”, motility loci were organized by motility locus/gene context. CDSs without a locus tag were grouped together and not with the other CDSs of their respective motility loci (heat-plots B). The standard locus tags for *R. intestinalis* L1-82 and *R. inulinivorans* A2-194 have been shortened to “L182_” and “A2194_” respectively for the preparation of these heat-plots.
(PDF)

Figure S5 **Recruitment plots demonstrating the presence or absence of the flagellin proteins of interest in 27 metagenomes.** A: Community dwelling individuals. B: Individuals from rehabilitation (EM219-EM238) and long-stay (EM173-EM308) community settings. Each plot shows matches with 80–100% similarity to the query flagellin sequence, which are labelled with locus tags. Matches in red are in the same orientation as the query sequence. Matches in blue are inverted relative to the query sequence. No matches were detected for four long-stay individuals, EM208, EM227, EM238 or EM275, so no plots could be constructed.
(PDF)

Table S1 **Locus tags for motility loci from genomes of interest.**
(DOC)

Table S2 **Amino-terminal sequences of** ***E. rectale*** **A1-86 and** ***R. inulinivorans*** **A2-194 flagellin proteins.**
(DOC)

Table S3 **Relative abundance (%) of each target species in 25 of the shotgun metagenomes of interest, as calculated by MetaPhlAn.**
(DOC)

Table S4 **Estimated target genome coverage in each metagenome.**
(DOC)

Table S5 **Summary of the number of ORFs per assembled metagenome identified as a motility gene or gene fragment from a species of interest.**
(DOC)

Table S6 **“Cell motility” COG category analysis for assembled metagenomes.**
(DOC)

Table S7 **Description of COGs within Cell Motility Category N.**
(DOC)

Table S8 **Strains and genomes used in this study.**
(DOC)
