# Supplementary figures and images for: Pro-Inflammatory Flagellin Proteins of Prevalent Motile Commensal Bacteria Are Variably Abundant in the Intestinal Microbiome of Elderly Humans
Source: PLoS One. 2013 Jul 23;8(7):e68919. doi: 10.1371/journal.pone.0068919 (PMC3720852; doi:10.1371/journal.pone.0068919)

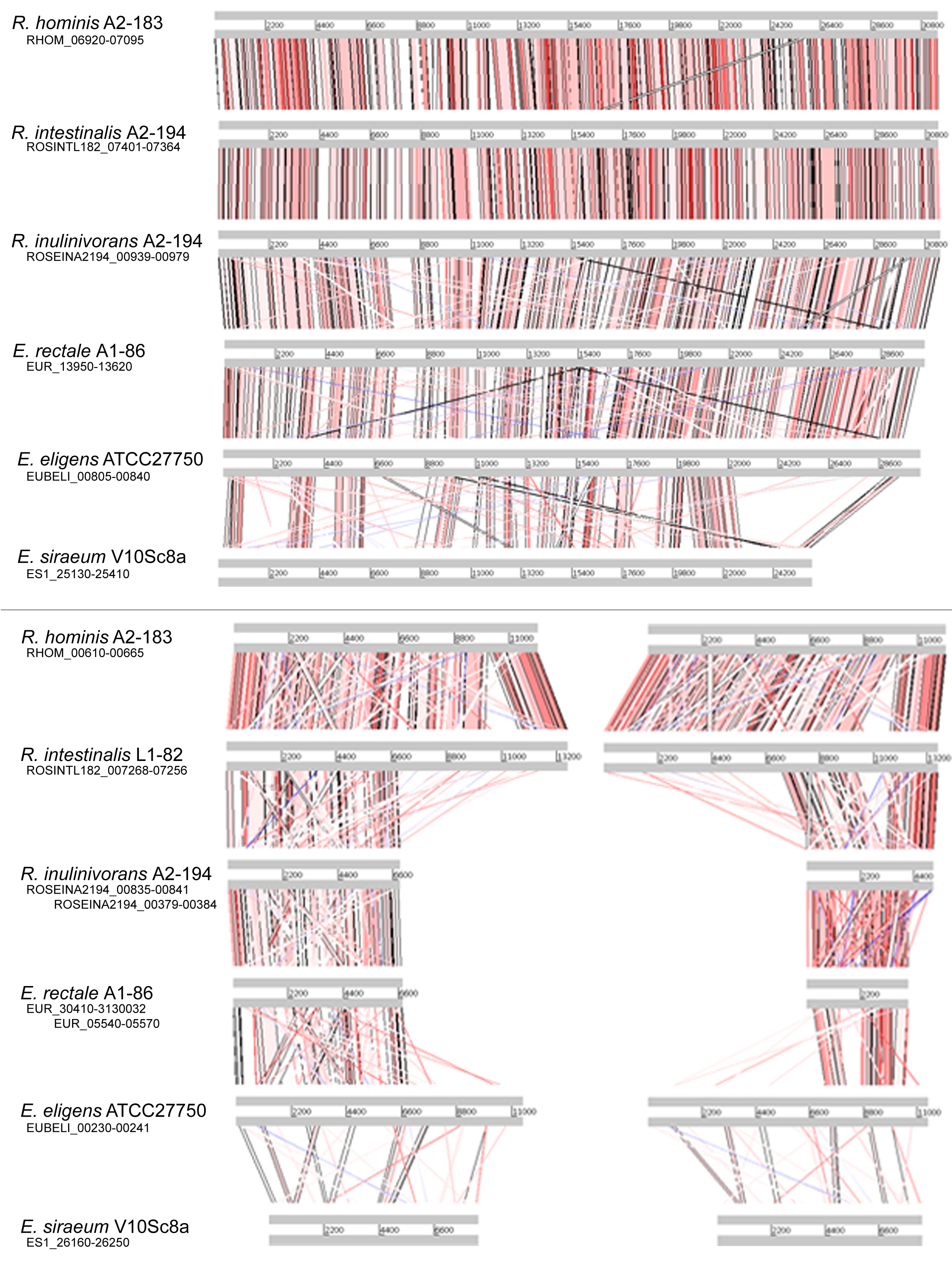

Supplement: Figure S1 — ACT alignments of flgB-fliA (top) and flgM-flgN/fliC (bottom) motility loci. Locus tags indicate which genomic region is represented. A minimum threshold of 30% identity was imposed on the alignments. Alignments involving E. rectale and R. inulinivorans flgM-csrA and flaG-flgN/fliC are on bottom left and right respectively. (TIF) [file pone.0068919.s001.tif]

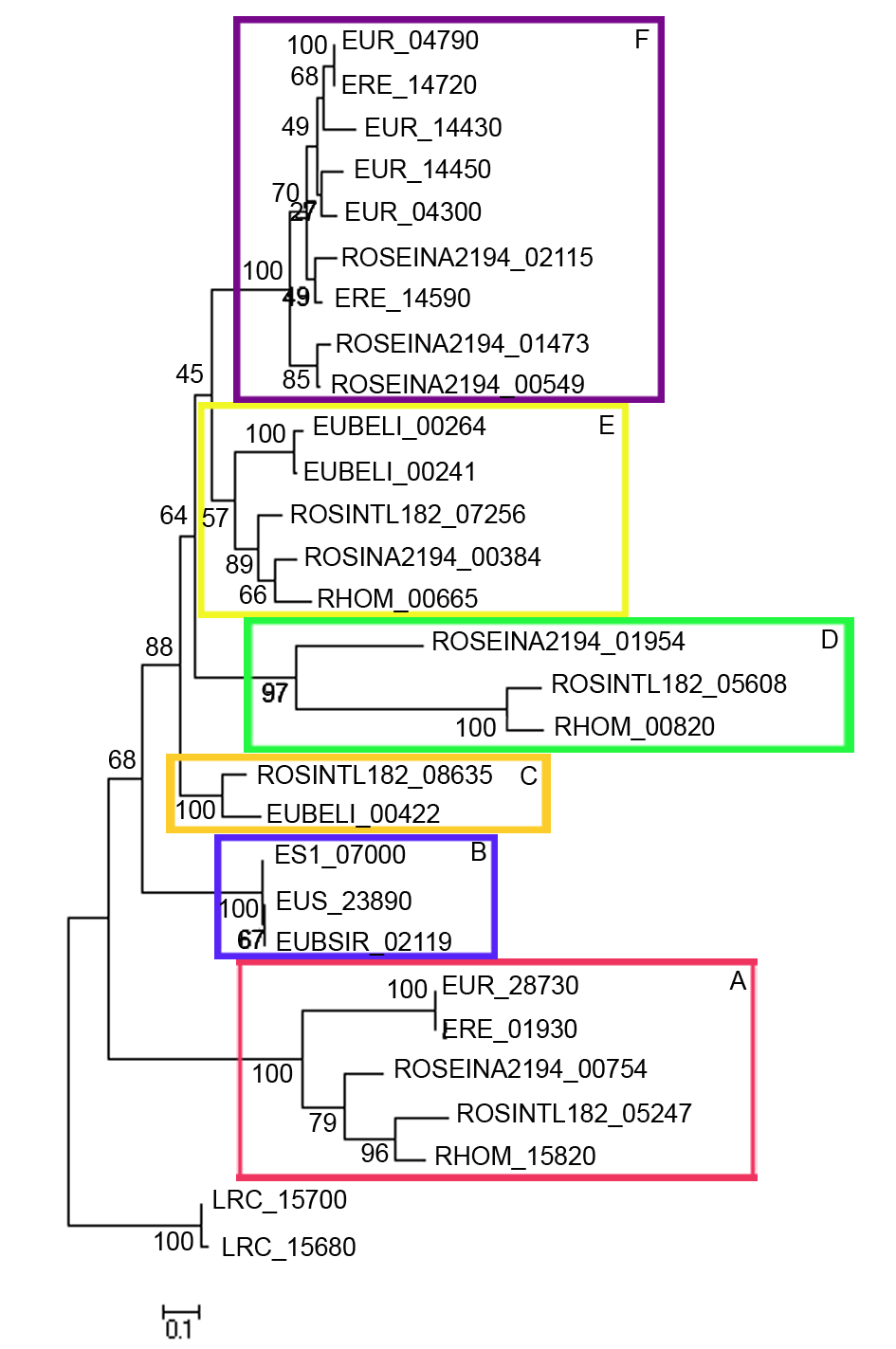

Supplement: Figure S2 — Phylogenetic tree of flagellin proteins. The flagellin tree was constructed from flagellin protein sequences using PHYML with model LG. Numbers at each node are bootstrap values. Locus tags were used to label flagellin proteins. Strongly supported clades (bootstrap ≥55) are surrounded by coloured boxes and are labelled with a letter A–F. ROSINTL182 = R. intestinalis L1-82, RHOM = R. hominis A2-183, ROSEINA2194 = R. inulinivorans A2-194, EUBELI = E. eligens ATCC27750, ES1 = E. siraeum V10Sc8a, EUBSIR = E. siraeum DSM15702, EUS = E. siraeum 70/3, EUR = E. rectale A1-86, ERE = E. rectale M104/1. (TIF) [file pone.0068919.s002.tif]

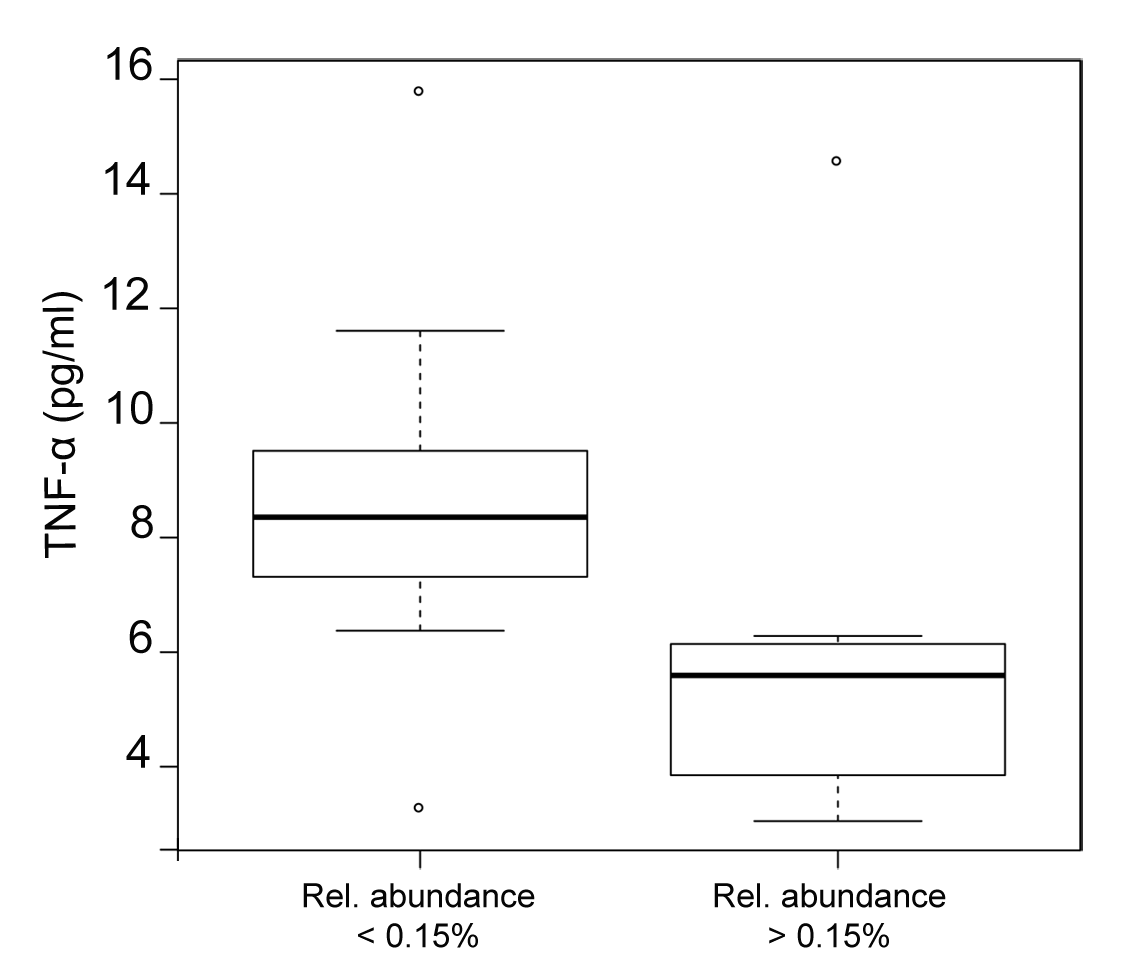

Supplement: Figure S3 — Association between E. siraeum relative abundance and serum TNF-α concentration. Boxplot showing median serum TNF-α concentration which is greater in individuals that harbor E. siraeum at <0.15% relative abundance (n = 14), than in individuals that harbor this organism at >0.15% relative abundance (n = 10). Boxplots show the median and interquartile range. Outliers are indicated by o symbols. Significance was assessed using the Spearman correlation coefficient. (TIF) [file pone.0068919.s003.tif]

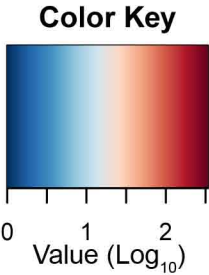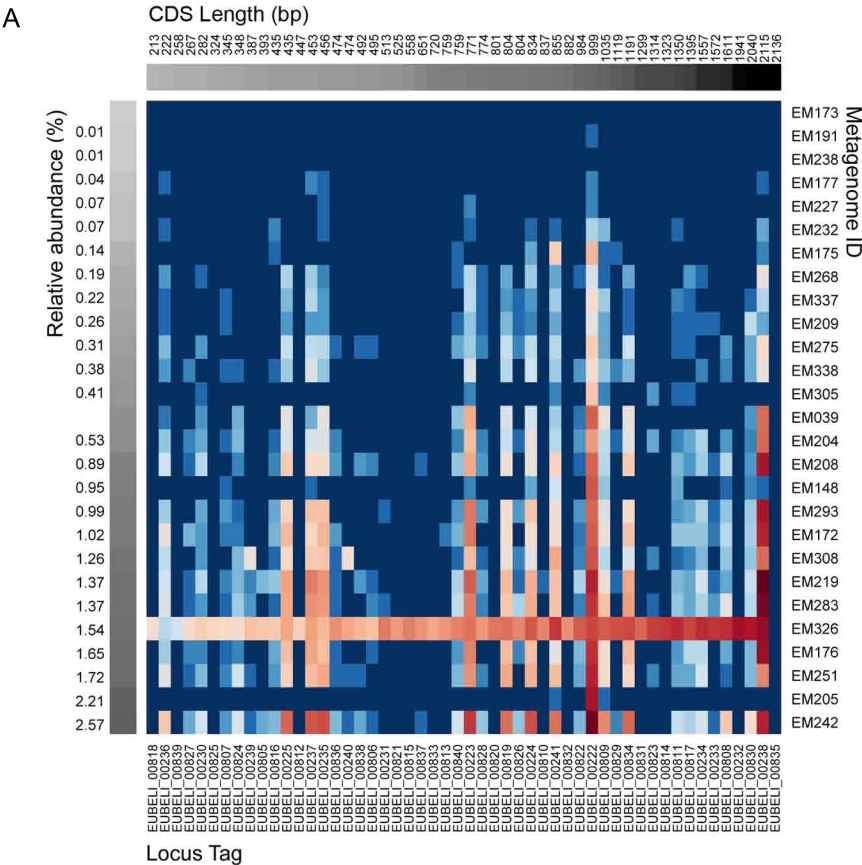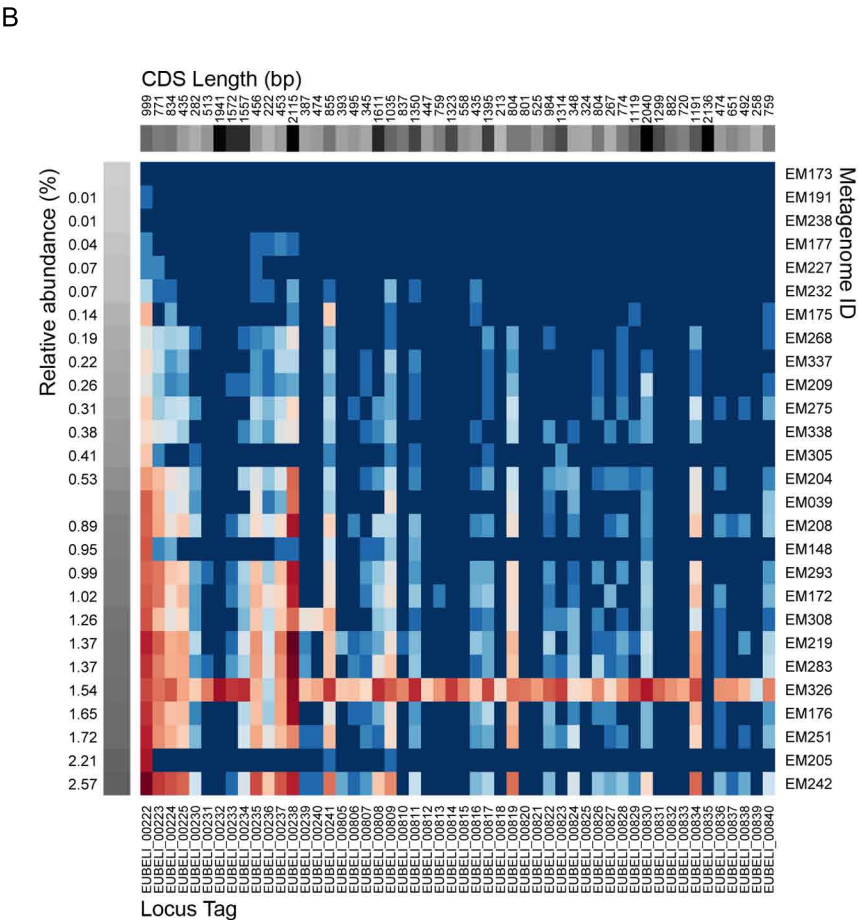

*E. rectale*

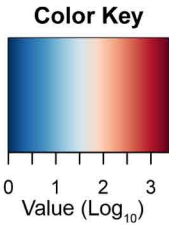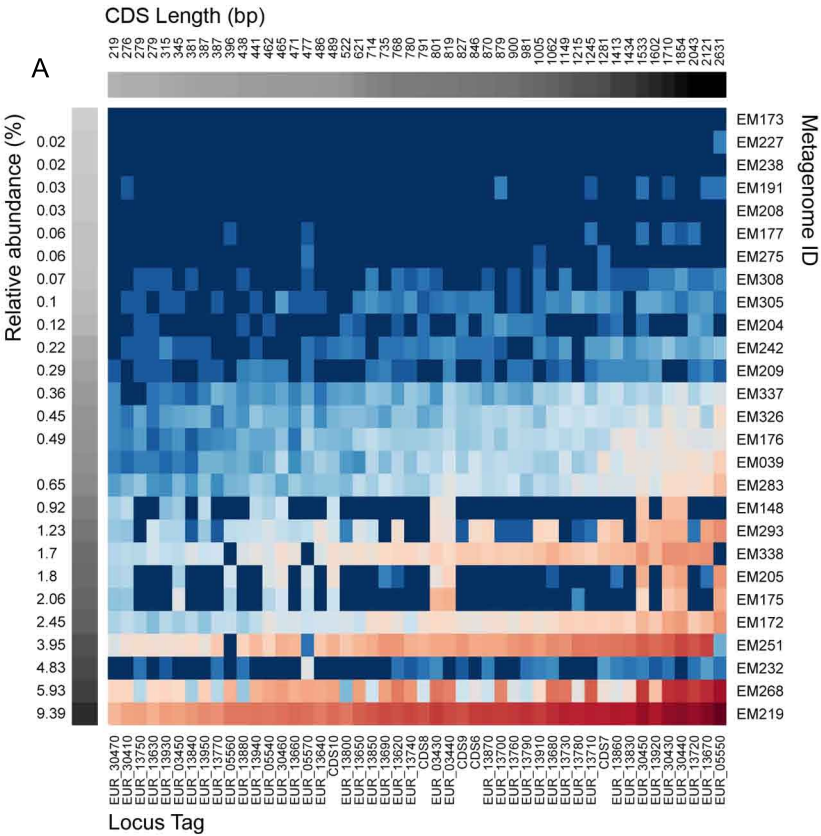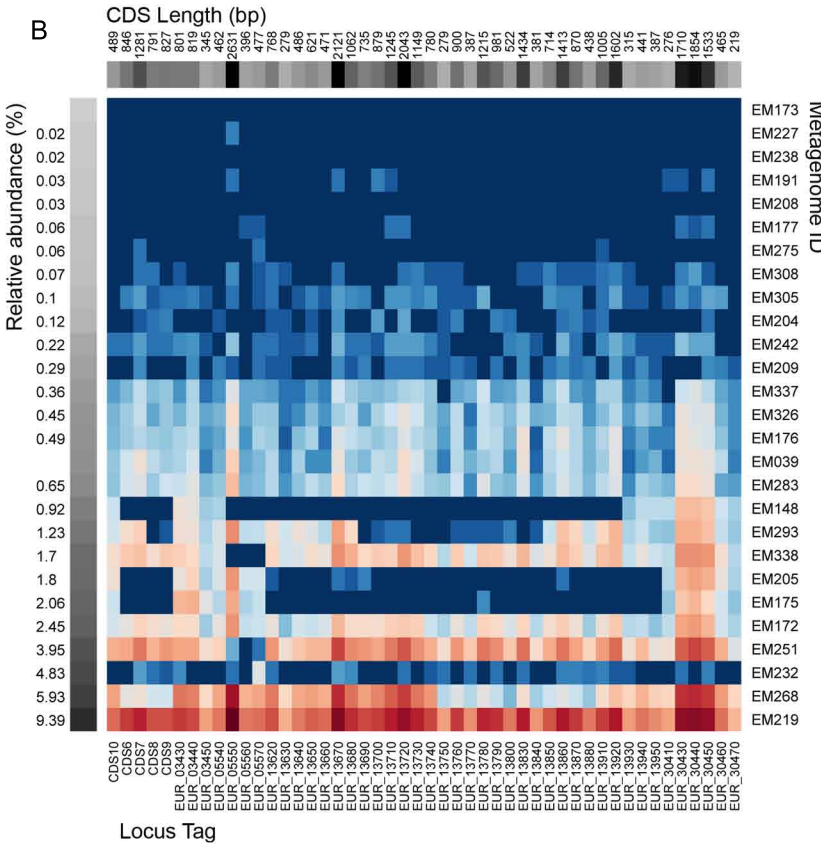

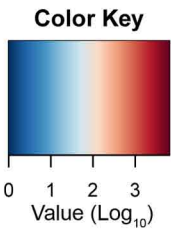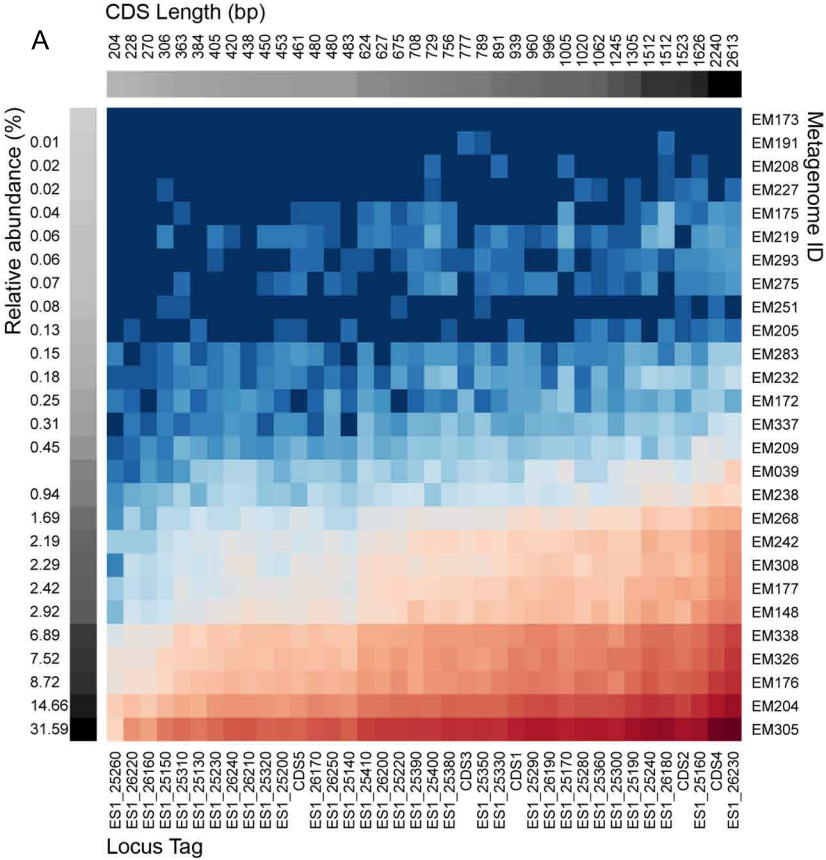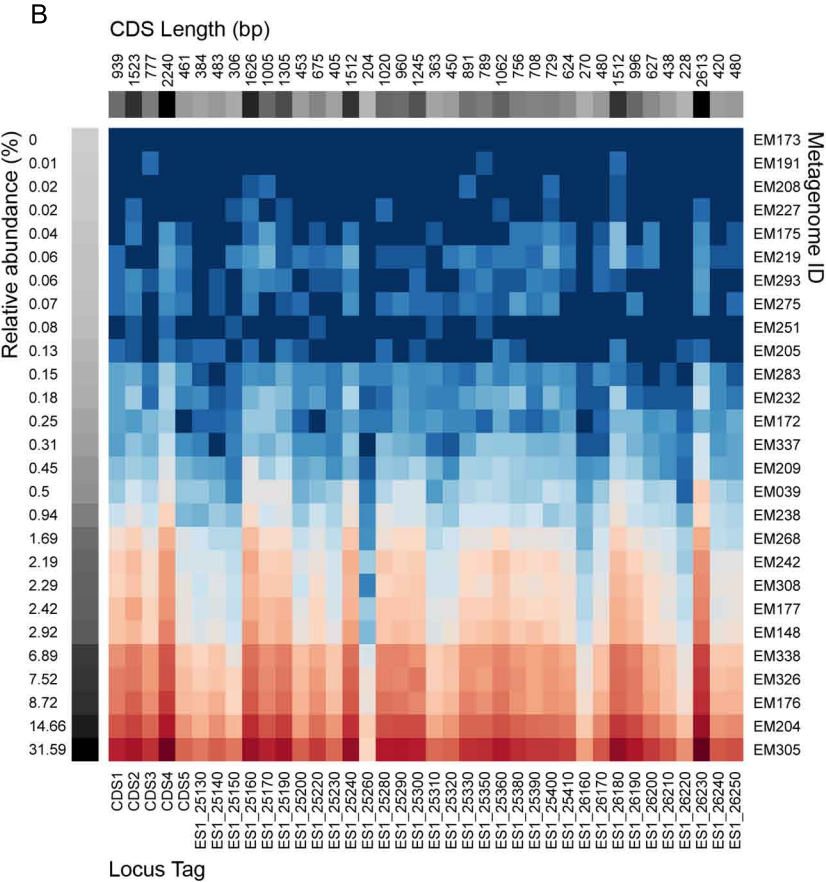

*R. intestinalis*

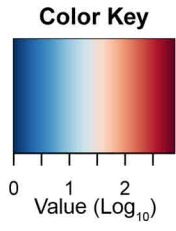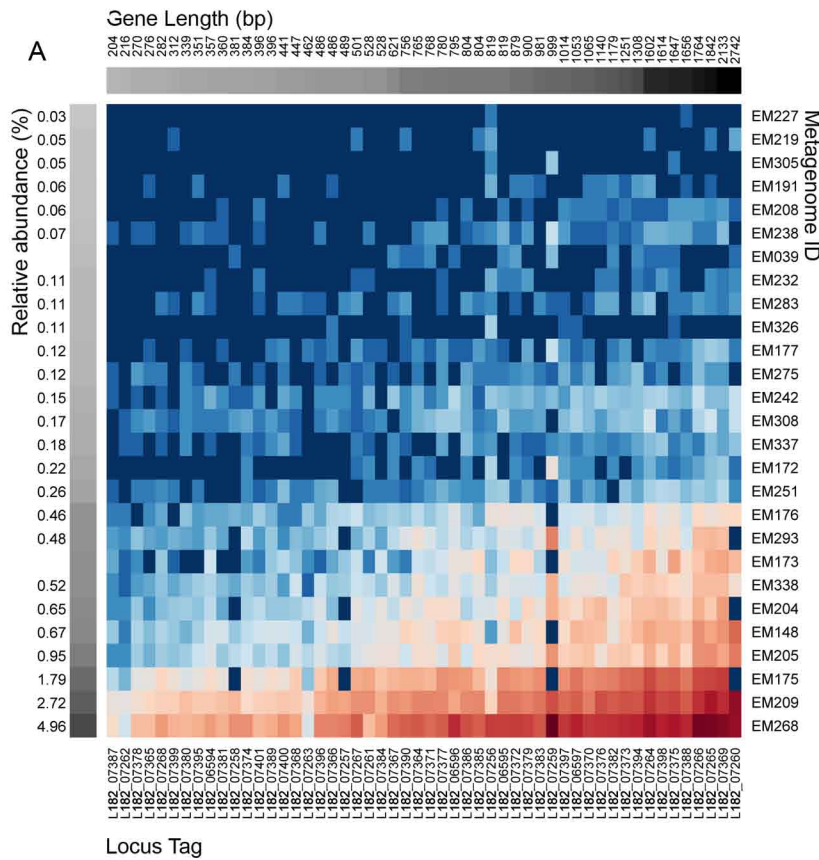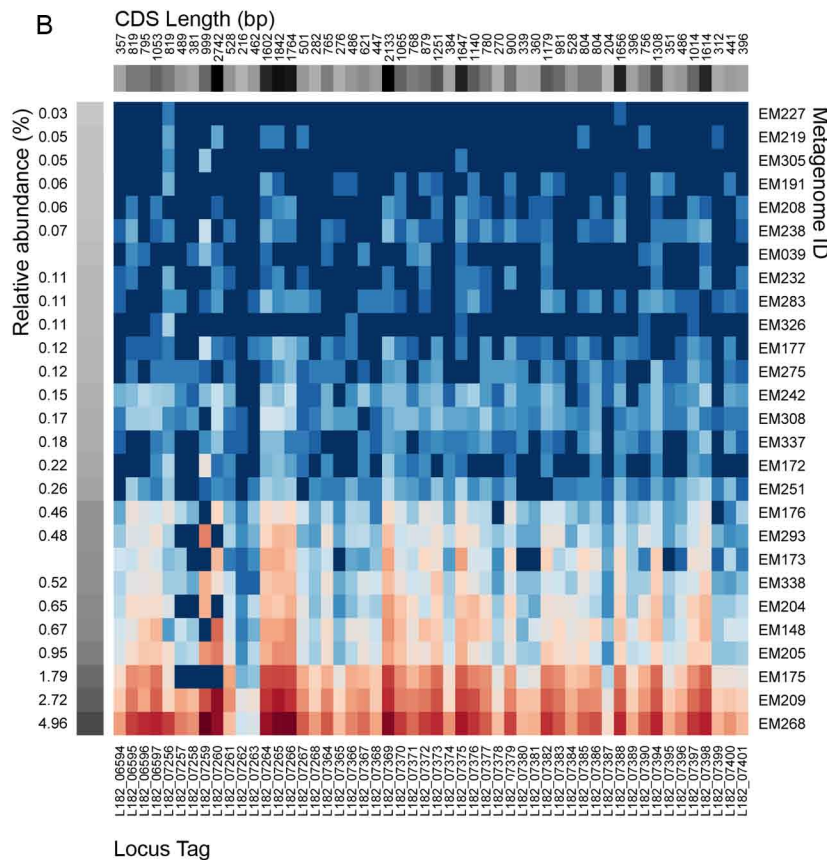

*R. inulinivorans*

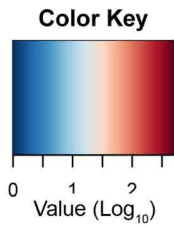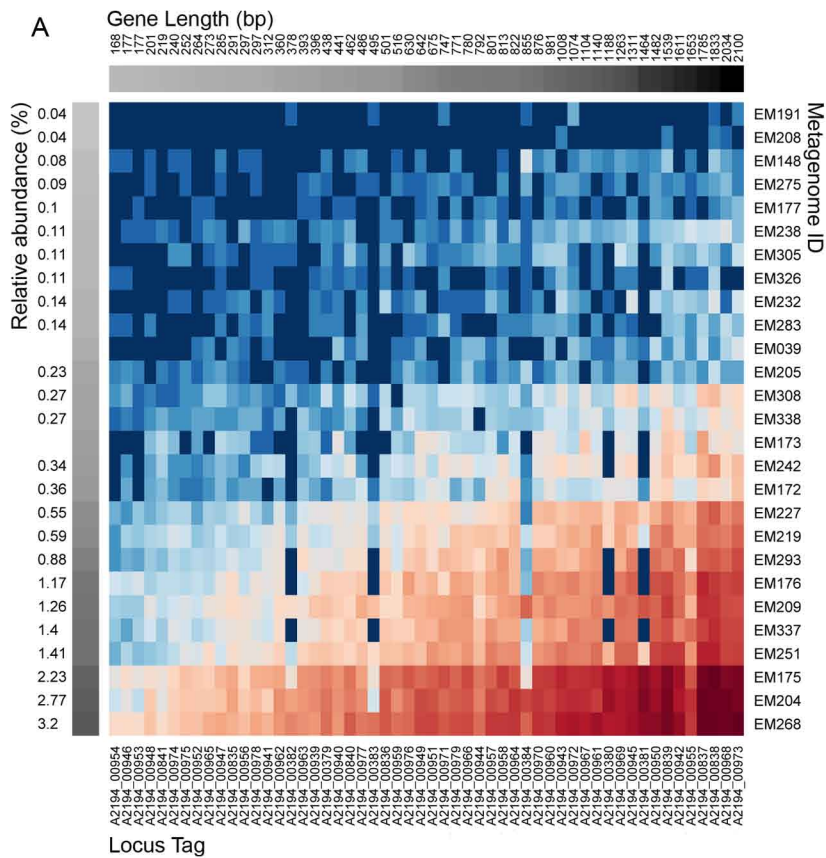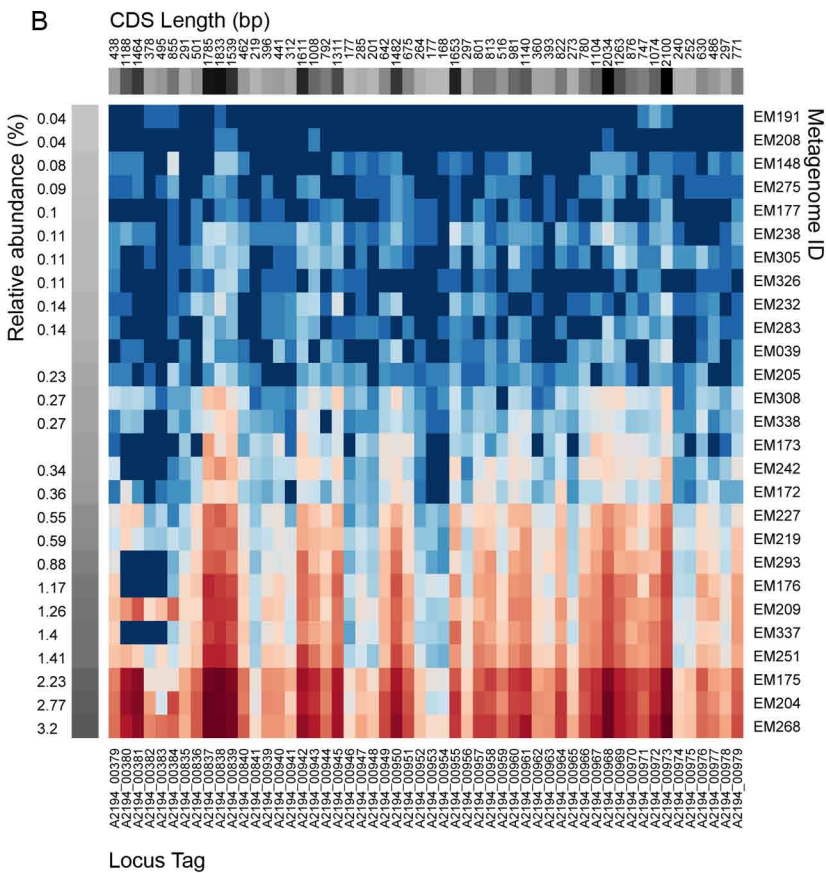

Supplement: Figure S4 — Heat-plots showing the relationship between the normalized number of reads mapped to target motility CDSs as a function of CDS length and target species relative abundance. Heat-plots labelled “A” show that the normalized number of reads that mapped to each target gene increases with increasing CDS length and species relative abundance. Heat-plots labelled “B” show that the normalized number of reads that mapped to target CDSs varied depending on gene context. For each species, heat-plots A and B present the same data, but differ due to alternative arrangements of the CDSs on the X axis. In heat-plots labelled “A”, CDSs are arranged according to increasing length, while in heat-plots labelled “B”, motility loci were organized by motility locus/gene context. CDSs without a locus tag were grouped together and not with the other CDSs of their respective motility loci (heat-plots B). The standard locus tags for R. intestinalis L1-82 and R. inulinivorans A2-194 have been shortened to “L182_” and “A2194_” respectively for the preparation of these heat-plots. (PDF) [file pone.0068919.s004.pdf]

A

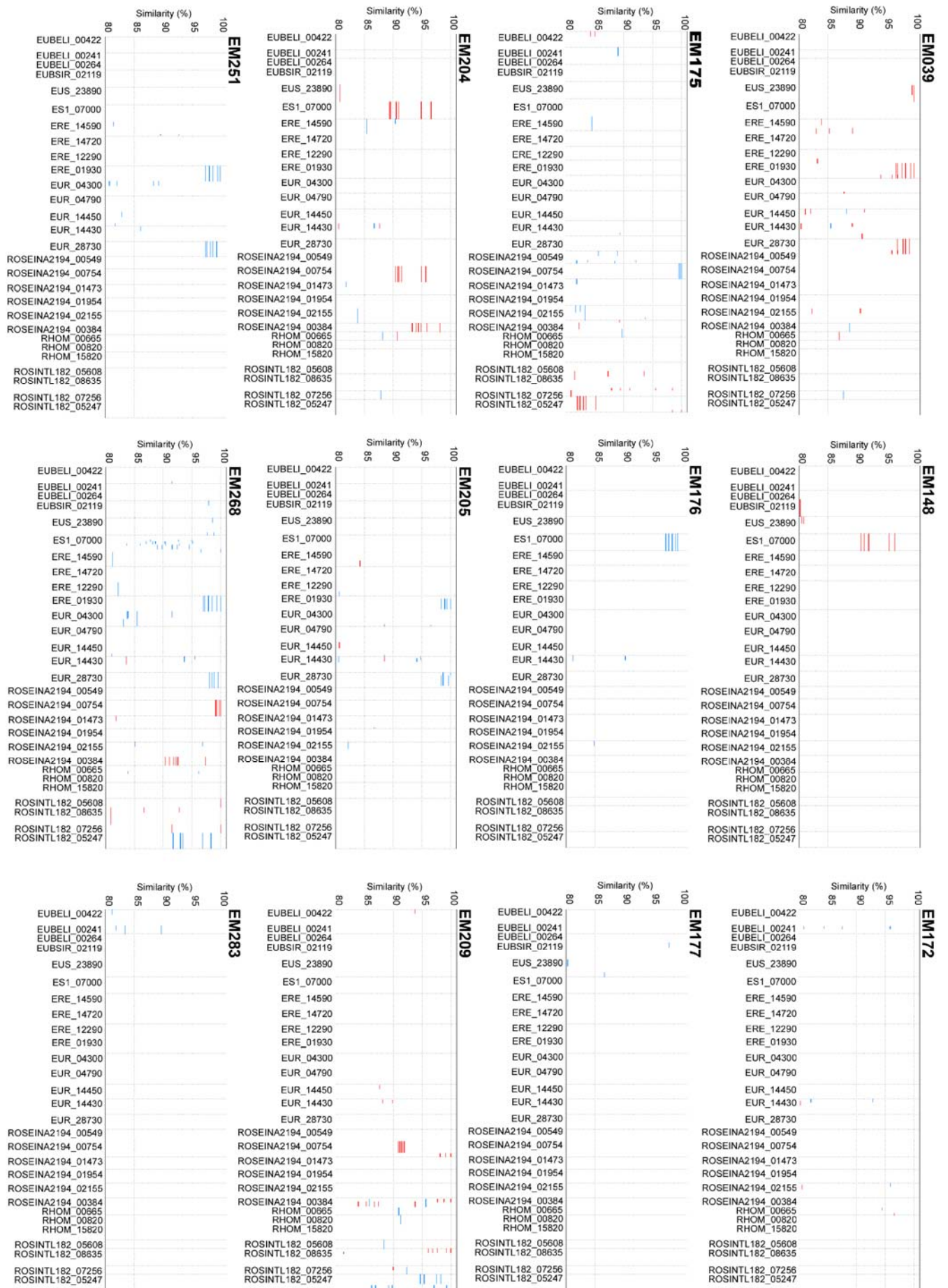

B

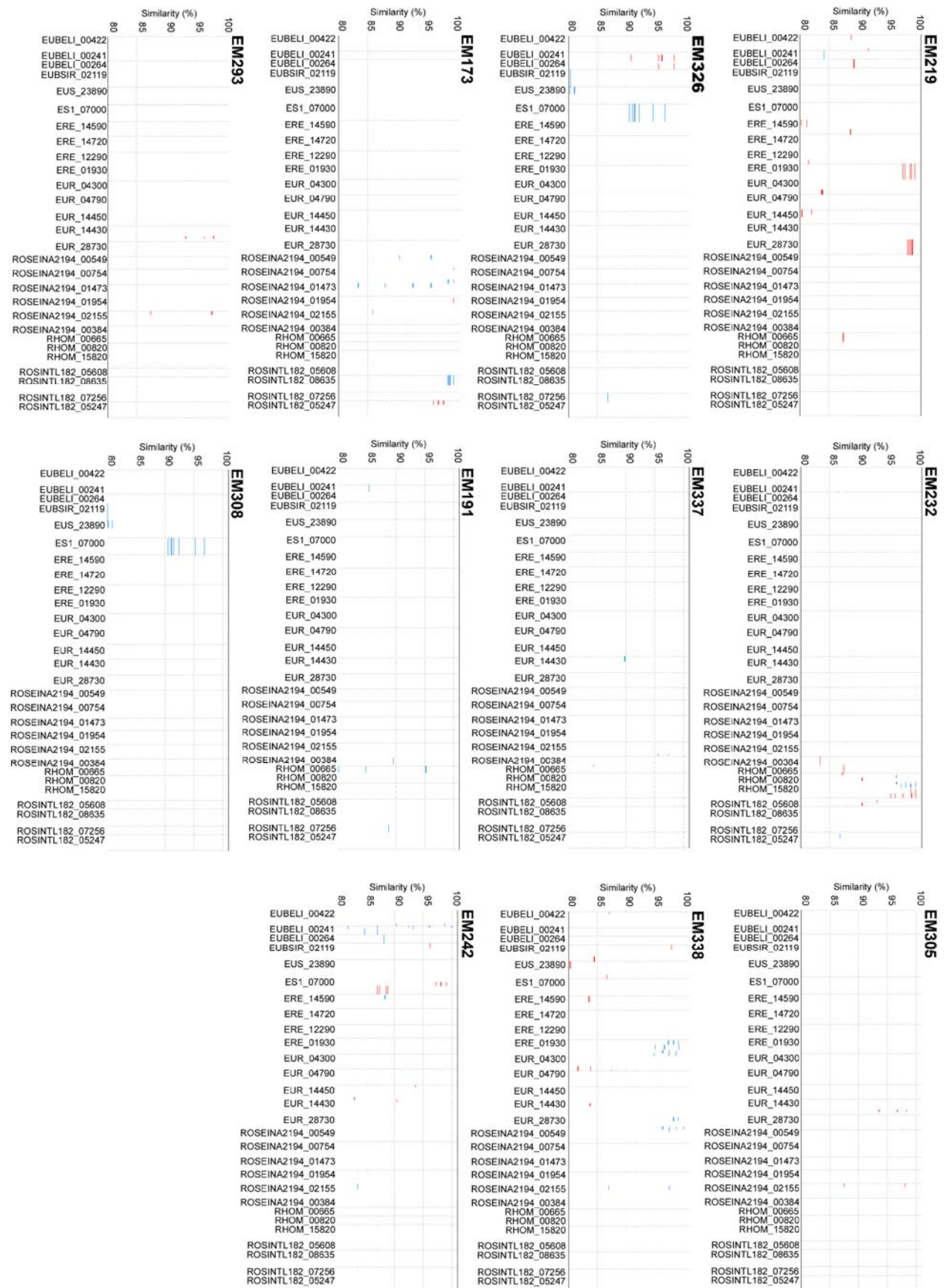

Supplement: Figure S5 — Recruitment plots demonstrating the presence or absence of the flagellin proteins of interest in 27 metagenomes. A: Community dwelling individuals. B: Individuals from rehabilitation (EM219-EM238) and long-stay (EM173-EM308) community settings. Each plot shows matches with 80–100% similarity to the query flagellin sequence, which are labelled with locus tags. Matches in red are in the same orientation as the query sequence. Matches in blue are inverted relative to the query sequence. No matches were detected for four long-stay individuals, EM208, EM227, EM238 or EM275, so no plots could be constructed. (PDF) [file pone.0068919.s005.pdf]
