# Supplementary material for: Pro-Inflammatory Flagellin Proteins of Prevalent Motile Commensal Bacteria Are Variably Abundant in the Intestinal Microbiome of Elderly Humans
Source: PLoS One. 2013 Jul 23;8(7):e68919. doi: 10.1371/journal.pone.0068919 (PMC3720852; doi:10.1371/journal.pone.0068919)
Supplement: Table S1 — Locus tags for motility loci from genomes of interest. (DOC) [file pone.0068919.s006.doc]

**Table S1: Locus tags for motility loci from genomes of interest.**

| **Protein** | ***E. rectale***  **A1-86** | | ***E. eligens* ATCC27750** | | ***R. intestinalis***  **L1-82** | | ***R. hominis***  **A2-183** | | ***R. inulinivorans***  **A2-194** | | | ***E. siraeum* V10Sc8a** |
| --- | --- | --- | --- | --- | --- | --- | --- | --- | --- | --- | --- | --- |
|  |  | |  | |  | |  | |  | | |  |
| **Locus A** | | | | | | | | | | | | |
|  |  | |  | |  | |  | |  | | |  |
| **FlgB** | EUR_13950 | | EUBELI_00805 | | ROSINTL182_07401 | | RHOM_06920 | | ROSEINA2194_00939 | | | ES1_25130 |
| **FlgC** | EUR_13940 | | EUBELI_00806 | | ROSINTL182_07400 | | RHOM_06925 | | ROSEINA2194_00940 | | | ES1_25140 |
| **FliE** | EUR_13930 | | EUBELI_00807 | | ROSINTL182_07399 | | RHOM_06930 | | ROSEINA2194_00941 | | | ES1_25150 |
| **FliF** | EUR_13920 | | EUBELI_00808 | | ROSINTL182_07398 | | Present | | ROSEINA2194_00942 | | | ES1_25160 |
| **FliG** | EUR_13910 | | EUBELI_00809 | | ROSINTL182_07397 | | RHOM_06945 | | ROSEINA2194_00943 | | | ES1_25170 |
| **FliH** | Present | | EUBELI_00810 | | ROSINTL182_07396-07395 | | RHOM_06950 | | ROSEINA2194_00944 | | | Present |
| **FliI** | Present | | EUBELI_00811 | | ROSINTL182_07394 | | RHOM_06955 | | ROSEINA2194_00945 | | | ES1_25190 |
| **FliJ** | EUR_13880 | | EUBELI_00812 | | ROSINTL182_07393 | | RHOM_06960 | | ROSINA2194_00946-00947 | | | ES1_25200 |
| **Hypothetical** | Absent | | Absent | | Absent | | Absent | | ROSEINA2194_00948 | | | Absent |
| **Hypothetical** | EUR_13870 | | EUBELI_00813 | | ROSINTL182_07392 | | RHOM_06965 | | ROSEINA2194_00949 | | | Absent |
| **FliK** | EUR_13860 | | EUBELI_00814 | | ROSINTL182_07391 | | RHOM_06970 | | ROSEINA2194_00950 | | | Present |
| **FlgD** | EUR_13850 | | EUBELI_00815 | | ROSINTL182_07390 | | RHOM_06975 | | ROSEINA2194_00951 | | | ES1_25220 |
| **Hypothetical** | Absent | | Absent | | Absent | | Absent | | ROSEINA2194_00952 | | | Absent |
| **FOP** | EUR_13840 | | EUBELI_00816 | | ROSINTL182_07389 | | RHOM_06980 | | ROSEINA2194_00953-00954 | | | ES1_25230 |
| **FlgE** | EUR_13830 | | EUBELI_00817 | | ROSINTL182_07388 | | RHOM_06985 | | ROSEINA2194_00955 | | | ES1_25240 |
| **FlbD** | Absent | | EUBELI_00818 | | ROSINTL182_07387 | | Absent | | ROSEINA2194_00956 | | | ES1_25260 |
| **MotA** | Present | | EUBELI_00819 | | ROSINTL182_07386 | | RHOM_06990 | | ROSEINA2194_00957 | | | Present |
| **MotB** | Present | | EUBELI_00820 | | ROSINTL182_07385 | | RHOM_06995 | | ROSEINA2194_00958 | | | ES1_25280 |
| **FliL** | EUR_13800 | | EUBELI_00821 | | ROSINTL182_07384 | | RHOM_07000 | | ROSEINA2194_00959 | | | Absent |
| **FliM** | EUR_13790 | | EUBELI_00822 | | ROSINTL182_07383 | | RHOM_07005 | | ROSEINA2194_00960 | | | ES1_25290 |
| **FliN** | EUR_13780 | | EUBELI_00823 | | ROSINTL182_07382 | | RHOM_07010 | | ROSEINA2194_00961 | | | ES1_25300 |
| **CheY-like** | Absent | | EUBELI_00824 | | ROSINTL182_07381 | | RHOM_07015 | | ROSEINA2194_00962 | | | ES1_25310 |
| **FliO** | EUR_13770 | | EUBELI_00825 | | ROSINTL182_07380 | | RHOM_07020 | | ROSEINA2194_00963 | | | ES1_25320 |
| **FliP** | EUR_13760 | | EUBELI_00826 | | ROSINTL182_07379 | | RHOM_07025 | | ROSEINA2194_00964 | | | ES1_25330 |
| **FliQ** | EUR_13750 | | EUBELI_00827 | | ROSINTL182_07378 | | RHOM_07030 | | ROSEINA2194_00965 | | | Present |
| **FliR** | EUR_13740 | | EUBELI_00828 | | ROSINTL182_07377 | | RHOM_07035 | | ROSEINA2194_00966 | | | ES1_25350 |
| **FlhB** | EUR_13730 | | EUBELI_00829 | | ROSINTL182_07376 | | RHOM_07040 | | ROSEINA2194_00967 | | | ES1_25360 |
| **FlhA** | EUR_13720 | | EUBELI_00830 | | ROSINTL182_07375-07374 | | RHOM_07045 | | ROSEINA2194_00968 | | | Present |
| **FlhF** | EUR_13710 | | EUBELI_00831 | | ROSINTL182_07373 | | RHOM_07050 | | ROSEINA2194_00969 | | | Absent |
| **FlhG** | EUR_13700 | | EUBELI_00832 | | ROSINTL182_07372 | | RHOM_07055 | | ROSEINA2194_00970 | | | Absent |
| **PilZ** | EUR_13690 | | EUBELI_00833 | | ROSINTL182_07371 | | RHOM_07060 | | ROSEINA2194_00971 | | | Absent |
| **CheB** | EUR_13680 | | EUBELI_00834 | | ROSINTL182_07370 | | RHOM_07065 | | ROSEINA2194_00972 | | | Absent |
| **CheA** | EUR_13670 | | EUBELI_00835 | | ROSINTL182_07369 | | RHOM_07070 | | ROSEINA2194_00973 | | | Absent |
| **CheW** | EUR_13660 | | EUBELI_00836 | | ROSINTL182_07368 | | RHOM_07075 | | ROSEINA2194_00974 | | | Absent |
| **CheC** | EUR_13650 | | EUBELI_00837 | | ROSINTL182_07367 | | RHOM_07080 | | ROSEINA2194_00976 | | | ES1_25410 |
| **CheD** | EUR_13640 | | EUBELI_00838 | | ROSINTL182_07366 | | RHOM_07085 | | ROSEINA2194_00977 | | | ES1_25420* |
| **Hypo** | EUR_13630 | | EUBELI_00839 | | ROSINTL182_07365 | | RHOM_07090 | | ROSEINA2194_00978 | | | Absent |
| **FliA** | EUR_13620 | | EUBELI_00840 | | ROSINTL182_07364 | | RHOM_07095 | | ROSEINA2194_00979 | | | ES1_25380 |
|  |  | |  | |  | |  | |  | | |  |
| **Accession No.** | FP929042.1 | | NC_012788.1 | | ABYJ02000109.1 ABYJ02000108.1 | | CP003040 | | ACFY01000039.1 | | | FP929059.1 |
| **TOTAL LENGTH (nt)** | 30520 | | 31143 | | 31347 | | 31496 | | 31444 | | | 26329 |
| **TOTAL CDS** | 34 | | 36 | | 36 | | 36 | | 38 | | | 29 |
|  |  | |  | |  | |  | |  | | |  |
|  | | | | | | | | | | | | |
| **Protein** | | ***E. rectale***  **A1-86** | | ***E. eligens* ATCC27750** | | ***R. intestinalis***  **L1-82** | | ***R. hominis* A2-183** | | ***R. inulinivorans***  **A2-194** | ***E. siraeum* V10Sc8a** | |
|  | | | | | | | | | | | | |
| **Locus B** | | | | | | | | | | | | |
|  |  | |  | |  | |  | |  | | |  |
| **MBL** | Absent | | EUBELI_00222 | | ROSINTL182_06597 | | RHOM_12465 | | ROSEINA2194_03009 | | | - |
| **FlgF** | EUR_03430 | | EUBELI_00223 | | ROSINTL182_06596 | | RHOM_12460 | | ROSEINA2194_03007-03008 | | | ES1_25390 |
| **FlgG** | EUR_03440 | | EUBELI_00224 | | ROSINTL182_06595 | | RHOM_12455 | | ROSEINA2194_03006 | | | ES1_25400 |
| **FlgJ** | EUR_03450 | | EUBELI_00225 | | ROSINTL182_06594 | | RHOM_12450 | | ROSEINA2194_03005 | | | - |
|  |  | |  | |  | |  | |  | | |  |
| **Accession No.** | FP929042.1 | | NC_012788.1 | | NZ_ABYJ02000069.1 | | NC_015977 | | ACFY01000115 | | |  |
| **TOTAL LENGTH (nt)** | 1984 | | 3195 | | 3220 | | 3158 | | 3119 | | |  |
| **TOTAL CDS** | 3 | | 4 | | 4 | | 4 | | 5 (4) | | |  |
|  |  | |  | |  | |  | |  | | |  |
| **Locus C** | | | | | | | | | | | | |
|  |  | |  | |  | |  | |  | | |  |
| **FlaG** | EUR_05540 | | EUBELI_00237 | | ROSINTL182_07261 | | RHOM_00645 | | ROSEINA2194_00379 | | | Absent |
| **FliD** | EUR_05550 | | EUBELI_00238 | | ROSINTL182_07260 | | RHOM_00650 | | ROSEINA2194_00380-00381 | | | ES1_26230 |
| **Transposase** | Absent | | Absent | | ROSINTL182_07259 | | Absent | | Absent | | | Absent |
| **FliS** | EUR_05560 | | EUBELI_00239 | | ROSINTL182_07258 | | RHOM_00655 | | ROSEINA2194_00382 | | | ES1_26240 |
| **FlgN** | EUR_05570 | | EUBELI_00240 | | ROSINTL182_07257 | | RHOM_00660 | | ROSEINA2194_00383 | | | ES1_26250 |
| **Flagellin** | Absent | | EUBELI_00241 | | ROSINTL182_07256 | | RHOM_00665 | | ROSEINA2194_00384 | | | Absent |
|  |  | |  | |  | |  | |  | | |  |
| **Accession No.** | FP929042.1 | | NC_012788.1 | | ABYJ02000104.1 | | CP003040 | | ACFY01000000 | | | FP929059.1 |
| **TOTAL LENGTH (nt)** | 6764 & 4152 | | 11440 | | 13616 | | 12130 | | 6871 & 5165 | | | 8351 |
| **TOTAL CDS** | 7 & 4 | | 12 | | 13 | | 12 | | 7 & 5 | | | 10 |

* Locus tag inferred based on its position immediately after ES1_25410.
