# Supplementary material for: Pro-Inflammatory Flagellin Proteins of Prevalent Motile Commensal Bacteria Are Variably Abundant in the Intestinal Microbiome of Elderly Humans
Source: PLoS One. 2013 Jul 23;8(7):e68919. doi: 10.1371/journal.pone.0068919 (PMC3720852; doi:10.1371/journal.pone.0068919)
Supplement: Table S2 — Amino-terminal sequences of E. rectale A1-86 and R. inulinivorans A2-194 flagellin proteins. (DOC) [file pone.0068919.s007.doc]

**Table S2: Amino-terminal sequences of *E. rectale* A1-86 and *R. inulinivorans* A2-194 flagellin proteins.**

| **Flagellin source & Size** | **1** | **2** | **3** | **4** | **5** | **6** | **7** | **8** | **9** | **10** |
| --- | --- | --- | --- | --- | --- | --- | --- | --- | --- | --- |
| *E. rectale*  ~ 52 kDa | M | V | V | Q | H | N | M | Q | A | A |
| *R. inulinivorans* ~ 51 kDa | M | V | V | Q | H | N | M | Q | A | A |
| *R. inulinivorans* ~ 43 kDa | M | V | V | Q | H | N | M | Q | A | A/M |
| *R. inulinivorans* ~ 29 kDa | M | V/Q | V/P | Q | H | N | N/M | T/M | A/T | A |
