# Supplementary material for: Pro-Inflammatory Flagellin Proteins of Prevalent Motile Commensal Bacteria Are Variably Abundant in the Intestinal Microbiome of Elderly Humans
Source: PLoS One. 2013 Jul 23;8(7):e68919. doi: 10.1371/journal.pone.0068919 (PMC3720852; doi:10.1371/journal.pone.0068919)
Supplement: Table S3 — Relative abundance (%) of each target species in 25 of the shotgun metagenomes of interest, as calculated by MetaPhlAn. (DOC) [file pone.0068919.s008.doc]

**Table S3: Relative abundance (%) of each target species in 25 of the shotgun metagenomes of interest, as calculated by MetaPhlAn.**

| **Metagenome** | **Location** | ***E. eligens*** | | ***E. rectale*** | | ***E. siraeum*** | | ***R. intestinalis*** | | ***R. inulinivorans*** | |
| --- | --- | --- | --- | --- | --- | --- | --- | --- | --- | --- | --- |
| EM148 | Community |  | 0.95 |  | 0.92 |  | 2.92 |  | 0.67 |  | 0.08 |
| EM172 | Community |  | 1.02 |  | 2.45 |  | 0.25 |  | 0.22 |  | 0.36 |
| EM175 | Community |  | 0.14 |  | 2.06 |  | 0.04 |  | 1.79 |  | 2.23 |
| EM176 | Community |  | 1.65 |  | 0.49 |  | 8.72 |  | 0.46 |  | 1.17 |
| EM177 | Community |  | 0.04 |  | 0.06 |  | 2.42 |  | 0.12 |  | 0.1 |
| EM204 | Community |  | 0.53 |  | 0.12 |  | 14.66 |  | 0.65 |  | 2.77 |
| EM205 | Community |  | 2.21 |  | 1.8 |  | 0.13 |  | 0.95 |  | 0.23 |
| EM209 | Community |  | 0.26 |  | 0.29 |  | 0.45 |  | 2.72 |  | 1.26 |
| EM251 | Community |  | 1.72 |  | 3.95 |  | 0.08 |  | 0.26 |  | 1.41 |
| EM268 | Community |  | 0.19 |  | 5.93 |  | 1.69 |  | 4.96 |  | 3.2 |
| EM283 | Community |  | 1.37 |  | 0.65 |  | 0.15 |  | 0.11 |  | 0.14 |
| EM219 | Rehabilitation |  | 1.37 |  | 9.39 |  | 0.06 |  | 0.05 |  | 0.59 |
| EM232 | Rehabilitation |  | 0.07 |  | 4.83 |  | 0.18 |  | 0.11 |  | 0.14 |
| EM305 | Rehabilitation |  | 0.41 |  | 0.1 |  | 31.59 |  | 0.05 |  | 0.11 |
| EM326 | Rehabilitation |  | 1.54 |  | 0.45 |  | 7.52 |  | 0.11 |  | 0.11 |
| EM337 | Rehabilitation |  | 0.22 |  | 0.36 |  | 0.31 |  | 0.18 |  | 1.4 |
| EM338 | Rehabilitation |  | 0.38 |  | 1.7 |  | 6.89 |  | 0.52 |  | 0.27 |
| EM191 | Longstay |  | 0.01 |  | 0.03 |  | 0.01 |  | 0.06 |  | 0.04 |
| EM208 | Longstay |  | 0.89 |  | 0.03 |  | 0.02 |  | 0.06 |  | 0.04 |
| EM227 | Longstay |  | 0.07 |  | 0.02 |  | 0.02 |  | 0.03 |  | 0.55 |
| EM238 | Longstay |  | 0.01 |  | 0.02 |  | 0.94 |  | 0.07 |  | 0.11 |
| EM242 | Longstay |  | 2.57 |  | 0.22 |  | 2.19 |  | 0.15 |  | 0.34 |
| EM275 | Longstay |  | 0.31 |  | 0.06 |  | 0.07 |  | 0.12 |  | 0.09 |
| EM293 | Longstay |  | 0.99 |  | 1.23 |  | 0.06 |  | 0.48 |  | 0.88 |
| EM308 | Longstay |  | 1.26 |  | 0.07 |  | 2.29 |  | 0.17 |  | 0.27 |

Note: Due to different assembly procedures, EM039 and EM173 were excluded from MetaPhlAn analysis.
