# Supplementary material for: Pro-Inflammatory Flagellin Proteins of Prevalent Motile Commensal Bacteria Are Variably Abundant in the Intestinal Microbiome of Elderly Humans
Source: PLoS One. 2013 Jul 23;8(7):e68919. doi: 10.1371/journal.pone.0068919 (PMC3720852; doi:10.1371/journal.pone.0068919)
Supplement: Table S4 — Estimated target genome coverage in each metagenome. (DOC) [file pone.0068919.s009.doc]

**Table S4: Estimated target genome coverage in each metagenome.**

| **Metagenome** | **Species** | **Rel. Abundance target species (%)** | **Estimated coverage** |
| --- | --- | --- | --- |
| EM148 | *E. eligens* | 0.95 | 16.45 |
| 4795.11 Mb | *E. rectale* | 0.92 | 12.62 |
|  | *E. siraeum* | 2.92 | 49.53 |
|  | *R. intestinalis* | 0.67 | 7.52 |
|  | *R. inulinivorans* | 0.08 | 0.95 |
| EM172 | *E. eligens* | 1.02 | 16.47 |
| 4472.60 Mb | *E. rectale* | 2.45 | 31.34 |
|  | *E. siraeum* | 0.25 | 3.96 |
|  | *R. intestinalis* | 0.22 | 2.30 |
|  | *R. inulinivorans* | 0.36 | 3.98 |
| EM175 | *E. eligens* | 0.14 | 2.41 |
| 4772.91 Mb | *E. rectale* | 2.06 | 28.12 |
|  | *E. siraeum* | 0.04 | 0.68 |
|  | *R. intestinalis* | 1.79 | 20.01 |
|  | *R. inulinivorans* | 2.23 | 26.28 |
| EM176 | *E. eligens* | 1.65 | 28.48 |
| 4780.81 Mb | *E. rectale* | 0.49 | 6.70 |
|  | *E. siraeum* | 8.72 | 147.48 |
|  | *R. intestinalis* | 0.46 | 5.15 |
|  | *R. inulinivorans* | 1.17 | 13.81 |
| EM177 | *E. eligens* | 0.04 | 0.69 |
| 4776.79 Mb | *E. rectale* | 0.06 | 0.82 |
|  | *E. siraeum* | 2.42 | 40.90 |
|  | *R. intestinalis* | 0.12 | 1.34 |
|  | *R. inulinivorans* | 0.1 | 1.18 |
| EM204 | *E. eligens* | 0.53 | 9.14 |
| 4779.02 Mb | *E. rectale* | 0.12 | 1.64 |
|  | *E. siraeum* | 14.66 | 247.86 |
|  | *R. intestinalis* | 0.65 | 7.27 |
|  | *R. inulinivorans* | 2.77 | 32.69 |
| EM205 | *E. eligens* | 2.21 | 38.19 |
| 4786.83 Mb | *E. rectale* | 1.8 | 24.64 |
|  | *E. siraeum* | 0.13 | 2.20 |
|  | *R. intestinalis* | 0.95 | 10.65 |
|  | *R. inulinivorans* | 0.23 | 2.72 |
| EM209 | *E. eligens* | 0.26 | 4.49 |
| 4788.57 Mb | *E. rectale* | 0.29 | 3.97 |
|  | *E. siraeum* | 0.45 | 7.62 |
|  | *R. intestinalis* | 2.72 | 30.50 |
|  | *R. inulinivorans* | 1.26 | 14.90 |
| EM251 | *E. eligens* | 1.72 | 29.72 |
| 4786.59 Mb | *E. rectale* | 3.95 | 54.07 |
|  | *E. siraeum* | 0.08 | 1.35 |
|  | *R. intestinalis* | 0.26 | 2.91 |
|  | *R. inulinivorans* | 1.41 | 16.66 |
| EM268 | *E. eligens* | 0.19 | 3.29 |
| 4789.76 Mb | *E. rectale* | 5.93 | 81.23 |
|  | *E. siraeum* | 1.69 | 28.64 |
|  | *R. intestinalis* | 4.96 | 55.64 |
|  | *R. inulinivorans* | 3.2 | 37.84 |
| EM283 | *E. eligens* | 1.37 | 23.68 |
| 4788.08 Mb | *E. rectale* | 0.65 | 8.90 |
|  | *E. siraeum* | 0.15 | 2.54 |
|  | *R. intestinalis* | 0.11 | 1.23 |
|  | *R. inulinivorans* | 0.14 | 1.66 |
| EM219 | *E. eligens* | 1.37 | 23.72 |
| 4796.21 Mb | *E. rectale* | 9.39 | 128.80 |
|  | *E. siraeum* | 0.06 | 1.02 |
|  | *R. intestinalis* | 0.05 | 0.56 |
|  | *R. inulinivorans* | 0.59 | 6.99 |
| EM232 | *E. eligens* | 0.07 | 1.21 |
| 4794.20 Mb | *E. rectale* | 4.83 | 66.22 |
|  | *E. siraeum* | 0.18 | 3.05 |
|  | *R. intestinalis* | 0.11 | 1.24 |
|  | *R. inulinivorans* | 0.14 | 1.66 |
| EM305 | *E. eligens* | 0.41 | 7.09 |
| 4793.44 Mb | *E. rectale* | 0.1 | 1.37 |
|  | *E. siraeum* | 31.59 | 535.70 |
|  | *R. intestinalis* | 0.05 | 0.56 |
|  | *R. inulinivorans* | 0.11 | 1.30 |
| EM326 | *E. eligens* | 1.54 | 26.66 |
| 4795.95 Mb | *E. rectale* | 0.45 | 6.17 |
|  | *E. siraeum* | 7.52 | 127.59 |
|  | *R. intestinalis* | 0.11 | 1.24 |
|  | *R. inulinivorans* | 0.11 | 1.30 |
| EM337 | *E. eligens* | 0.22 | 3.81 |
| 4798.09 Mb | *E. rectale* | 0.36 | 4.94 |
|  | *E. siraeum* | 0.31 | 5.26 |
|  | *R. intestinalis* | 0.18 | 2.02 |
|  | *R. inulinivorans* | 1.4 | 16.59 |
| EM338 | *E. eligens* | 0.38 | 6.58 |
| 4793.68 Mb | *E. rectale* | 1.7 | 23.31 |
|  | *E. siraeum* | 6.89 | 116.85 |
|  | *R. intestinalis* | 0.52 | 5.84 |
|  | *R. inulinivorans* | 0.27 | 3.20 |
| EM191 | *E. eligens* | 0.01 | 0.17 |
| 4789.62 Mb | *E. rectale* | 0.03 | 0.41 |
|  | *E. siraeum* | 0.01 | 0.17 |
|  | *R. intestinalis* | 0.06 | 0.67 |
|  | *R. inulinivorans* | 0.04 | 0.47 |
| EM208 | *E. eligens* | 0.89 | 15.39 |
| 4790.74 Mb | *E. rectale* | 0.03 | 0.41 |
|  | *E. siraeum* | 0.02 | 0.34 |
|  | *R. intestinalis* | 0.06 | 0.67 |
|  | *R. inulinivorans* | 0.04 | 0.47 |
| EM227 | *E. eligens* | 0.02 | 0.35 |
| 4794.62 Mb | *E. rectale* | 0.02 | 0.27 |
|  | *E. siraeum* | 0.02 | 0.34 |
|  | *R. intestinalis* | 0.03 | 0.34 |
|  | *R. inulinivorans* | 0.55 | 6.51 |
| EM238 | *E. eligens* | 0.01 | 0.17 |
| 4793.43 Mb | *E. rectale* | 0.02 | 0.27 |
|  | *E. siraeum* | 0.94 | 15.94 |
|  | *R. intestinalis* | 0.07 | 0.79 |
|  | *R. inulinivorans* | 0.11 | 1.30 |
| EM242 | *E. eligens* | 2.57 | 44.50 |
| 4796.50 Mb | *E. rectale* | 0.22 | 3.02 |
|  | *E. siraeum* | 2.19 | 37.16 |
|  | *R. intestinalis* | 0.15 | 1.68 |
|  | *R. inulinivorans* | 0.34 | 4.03 |
| EM275 | *E. eligens* | 0.31 | 5.37 |
| 4796.45 Mb | *E. rectale* | 0.06 | 0.82 |
|  | *E. siraeum* | 0.07 | 1.19 |
|  | *R. intestinalis* | 0.12 | 1.35 |
|  | *R. inulinivorans* | 0.09 | 1.07 |
| EM293 | *E. eligens* | 0.99 | 17.14 |
| 4795.73 Mb | *E. rectale* | 1.23 | 16.87 |
|  | *E. siraeum* | 0.06 | 1.02 |
|  | *R. intestinalis* | 0.48 | 5.39 |
|  | *R. inulinivorans* | 0.88 | 10.42 |
| EM308 | *E. eligens* | 1.26 | 21.81 |
| 4794.98 Mb | *E. rectale* | 0.07 | 0.96 |
|  | *E. siraeum* | 2.29 | 38.85 |
|  | *R. intestinalis* | 0.17 | 1.91 |
|  | *R. inulinivorans* | 0.27 | 3.20 |

Note: Average target genome sizes in Mb are as follows: *E. eligens* 2.27, *E. rectale*, 3.5, *E. siraeum* 2.83, *R. intestinalis*, 4.27, *R. inulinivorans*, 4.05.
