# Supplementary material for: Pro-Inflammatory Flagellin Proteins of Prevalent Motile Commensal Bacteria Are Variably Abundant in the Intestinal Microbiome of Elderly Humans
Source: PLoS One. 2013 Jul 23;8(7):e68919. doi: 10.1371/journal.pone.0068919 (PMC3720852; doi:10.1371/journal.pone.0068919)
Supplement: Table S5 — Summary of the number of ORFs per assembled metagenome identified as a motility gene or gene fragment from a species of interest. (DOC) [file pone.0068919.s010.doc]

**Table S3.5: Summary of the number of ORFs per assembled metagenome identified as a motility gene or gene fragment from a species of interest.**

| **Metagenome ID** | ***E. eligens*** | ***E. rectale*** | ***E. siraeum*** | ***R. hominis*** | ***R. intestinalis*** | ***R. inulinivorans*** |
| --- | --- | --- | --- | --- | --- | --- |
| **EM039** | 17 | 11 | 37 | - | - | - |
| **EM148** | - | - | 41 | - | - | - |
| **EM172** | 2 | 2 | - | - | - | - |
| **EM175** | - | - | - | - | 20 | 41 |
| **EM176** | - | - | 37 | - | - | - |
| **EM177** | - | - | 34 | - | - | - |
| **EM204** | - | - | 39 | - | - | 52 |
| **EM205** | - | 10 | - | - | 1 | 1 |
| **EM209** | - | - | - | - | 42 | 28 |
| **EM251** | - | 44 | - | - | - | - |
| **EM268** | - | 23 | 42 | - | 45 | 44 |
| **EM283** | 5 | - | - | - | - | - |
| **EM219** | - | 37 | - | - | - | - |
| **EM232** | - | - | - | 37 | - | - |
| **EM305** | - | - | 34 | - | - | - |
| **EM326** | 9 | - | 38 | - | - | - |
| **EM337** | - | - | - | 1 | - | 22 |
| **EM338** | - | 39 | 35 | - | - | - |
| **EM173** | - | - | - | - | 11 | 5 |
| **EM191** | - | - | - | 1 | - | - |
| **EM208** | - | - | - | - | - | - |
| **EM227** | - | - | - | - | - | - |
| **EM238** | - | - | - | - | - | - |
| **EM242** | - | - | 28 | - | - | - |
| **EM275** | - | - | - | - | - | - |
| **EM293** | 1 | - | - | - | - | - |
| **EM308** | - | - | 34 | - | - | - |
