# Supplementary material for: Pro-Inflammatory Flagellin Proteins of Prevalent Motile Commensal Bacteria Are Variably Abundant in the Intestinal Microbiome of Elderly Humans
Source: PLoS One. 2013 Jul 23;8(7):e68919. doi: 10.1371/journal.pone.0068919 (PMC3720852; doi:10.1371/journal.pone.0068919)
Supplement: Table S6 — “Cell motility” COG category analysis for assembled metagenomes. (DOC) [file pone.0068919.s011.doc]

| **Metagenome** | **MG-RAST**  **Accession Number** | **Total COGs represented** | **No. different "Cell Motility" COGS represented** | **Cell motility as % of total COGs** | **No. sequences in COG1344**  **(Flagellin and related hook-associated proteins)** |
| --- | --- | --- | --- | --- | --- |
| EM039 | 4491562.3 | 1893 | 13 | 0.69 | 12 |
| EM148 | 4491490.3 | 1431 | 5 | 0.35 | - |
| EM172 | 4491487.3 | 1808 | 12 | 0.66 | 14 |
| EM175 | 4491485.3 | 1754 | 9 | 0.51 | 7 |
| EM176 | 4491484.3 | 1731 | 14 | 0.81 | 29 |
| EM177 | 4491482.3 | 2026 | 12 | 0.59 | 4 |
| EM204 | 4495123.3 | 1674 | 7 | 0.42 | - |
| EM205 | 4491423.3 | 1836 | 16 | 0.87 | 23 |
| EM209 | 4491421.3 | 1765 | 9 | 0.51 | 11 |
| EM251 | 4491414.3 | 1520 | 11 | 0.72 | 7 |
| EM268 | 4491413.3 | 1671 | 10 | 0.60 | 8 |
| EM283 | 4491410.3 | 2235 | 19 | 0.85 | 16 |
| EM219 | 4491420.3 | 1834 | 9 | 0.49 | 4 |
| EM232 | 4491417.3 | 1764 | 7 | 0.40 | 3 |
| EM305 | 4491407.3 | 1839 | 14 | 0.76 | 8 |
| EM326 | 4491405.3 | 1717 | 15 | 0.87 | 7 |
| EM337 | 4491403.3 | 1972 | 16 | 0.81 | 8 |
| EM338 | 4491401.3 | 1921 | 15 | 0.78 | 11 |
| EM173 | 4491563.3 | 1777 | 9 | 0.51 | 7 |
| EM191 | 4491479.3 | 1770 | 13 | 0.73 | 3 |
| EM208 | 4491422.3 | 1535 | 9 | 0.59 | 2 |
| EM227 | 4491418.3 | 1554 | 2 | 0.13 | - |
| EM238 | 4491416.3 | 1661 | 3 | 0.18 | 1 |
| EM242 | 4491415.3 | 1545 | 9 | 0.58 | 12 |
| EM275 | 4491411.3 | 1817 | 7 | 0.39 | 1 |
| EM293 | 4491408.3 | 1503 | 5 | 0.33 | 3 |
| EM308 | 4491406.3 | 1820 | 8 | 0.44 | - |

**Table S6: “Cell Motility” COG category analysis for assembled metagenomes.**
