# Supplementary material for: Pro-Inflammatory Flagellin Proteins of Prevalent Motile Commensal Bacteria Are Variably Abundant in the Intestinal Microbiome of Elderly Humans
Source: PLoS One. 2013 Jul 23;8(7):e68919. doi: 10.1371/journal.pone.0068919 (PMC3720852; doi:10.1371/journal.pone.0068919)
Supplement: Table S7 — Description of COGs within Cell Motility Category N. (DOC) [file pone.0068919.s012.doc]

**Table S7: Description of COGs within Cell Motility Category N**.

Content from <http://www.ncbi.nlm.nih.gov/COG/grace/wiew.cgi?fun=N>.
