# Supplementary material for: Pro-Inflammatory Flagellin Proteins of Prevalent Motile Commensal Bacteria Are Variably Abundant in the Intestinal Microbiome of Elderly Humans
Source: PLoS One. 2013 Jul 23;8(7):e68919. doi: 10.1371/journal.pone.0068919 (PMC3720852; doi:10.1371/journal.pone.0068919)
Supplement: Table S8 — Strains and genomes used in this study. (DOC) [file pone.0068919.s013.doc]

**Table S8: Strains and genomes used in this study.**

| **Species** | **Strain** | **Isolated from:** | **Genome accession No.** | **No. Contigs** | **Contig N50 (nt)** | **No. Scaffolds** | **Scaffold N50 (nt)** | **Size (nt)** | **Genome Publication Reference (PMID)** | **Strain Publication Reference (PMID)** |
| --- | --- | --- | --- | --- | --- | --- | --- | --- | --- | --- |
| *E. eligens* | ATCC27750 | Human faeces | CP001104.1; CP001105.1; CP001106.1 | Complete Genome | - | 3 | - | 2,144,190; 60,455; 62,6744 | 19321416 | Holdeman and Moore, 1974 |
|  |  |  |  |  |  |  |  |  |  |  |
| *E. rectale* | A1-86 | Human faeces | FP929042.1 | n/a | n/a | n/a | n/a | 3,344,951 | Unpublished | 10742256 18599726 |
|  | M104/1 | GI tract | [FP929043.1](http://www.ncbi.nlm.nih.gov/nuccore/FP929043.1) | n/a | n/a | n/a | n/a | 3,698,419 | Unpublished | 15028695 |
|  |  |  |  |  |  |  |  |  |  |  |
| *E. siraeum* | V10Sc8a | Human faeces | [FP929059.1](http://www.ncbi.nlm.nih.gov/nuccore/FP929059.1) | n/a | n/a | n/a | n/a | 2,836,123 | Unpublished | SH Duncan & HJ Flint (Unpublished) |
|  | DSM_15702 | Human faeces | ABCA00000000.3 | 55 | 104,065 | 44 | 112,958 | 2,697,034 | Unpublished | Moore *et al.,* 1976 |
|  | 70/3 | Human faeces | [FP929044.1](http://www.ncbi.nlm.nih.gov/nuccore/FP929044.1) | n/a | n/a | n/a | n/a | 2,943,413 | Unpublished | SH Duncan & HJ Flint (Unpublished),  18537837 |
|  |  |  |  |  |  |  |  |  |  |  |
| *R. hominis* | A2-183 | Human faeces | CP003040.1 | Complete Genome | - | 1 | - | 3,592,125 | Unpublished | 10742256 |
|  |  |  |  |  |  |  |  |  |  |  |
| *R. intestinalis* | L1-82 | Human faeces | ABYJ00000000.2 | 409 | 29,464 | 102 | 123,125 | 4,411,375 | Unpublished | 10742256  12361264 |
|  |  |  |  |  |  |  |  |  |  |  |
| *R. inulinivorans* | A2-194 | Human faeces | ACFY01000000 | 179 | 57,343 | n/a | n/a | 4,048,462 | Unpublished | 10742256 |
|  |  |  |  |  |  |  |  |  |  |  |
